# Tuberculosis case notifications and outcomes in Peruvian prisons prior to and during the COVID-19 pandemic: a national-level interrupted time series analysis

**DOI:** 10.1016/j.lana.2024.100723

**Published:** 2024-03-27

**Authors:** Lena Faust, Guillermo Caceres-Cardenas, Leonardo Martinez, Sophie Huddart, Julia Rios Vidal, Ronald Corilloclla-Torres, Mayra Cordova Ayllon, Andrea Benedetti, Madhukar Pai, César Ugarte-Gil

**Affiliations:** a Department of Epidemiology, Biostatistics and Occupational Health, McGill University, Montréal, Canada; b McGill International TB Centre, McGill University, Montréal, Canada; c London School of Hygiene and Tropical Medicine, London, United Kingdom; d Instituto de Medicina Tropical Alexander von Humboldt, Universidad Peruana Cayetano Heredia, Lima, Perú; e School of Public Health, Boston University, Boston, USA; f Division of Pulmonary and Critical Care Medicine, University of California San Francisco, San Francisco, USA; g UCSF Center for Tuberculosis, University of California San Francisco, San Francisco, USA; h Dirección De Control y Prevención de la Tuberculosis (DPCT), Ministerio de Salud, Lima, Perú; i Subdireción de Salud Penitenciaria, Instituto Nacional Penitenciario, Lima, Perú; j Estrategia de Tuberculosis, Instituto Nacional Penitenciario, Lima, Perú; k School of Medicine, Universidad Peruana Cayetano Heredia, Lima, Perú; l Department of Epidemiology, School of Public and Population Health, University of Texas Medical Branch, Galveston, TX, USA

**Keywords:** Tuberculosis, Covid-19 pandemic, Epidemiology, Incarcerated populations, Peru

## Abstract

**Background:**

The COVID-19 pandemic has significantly disrupted tuberculosis (TB) programs, making it urgent to focus TB elimination efforts on key populations. People experiencing incarceration are at high risk for TB, however, how COVID-19-related disruptions have impacted incarcerated populations with TB is unknown.

**Methods:**

Using Peruvian National TB Program data from Jan 2018 to Dec 2021, an interrupted time series of drug-susceptible (DS) TB case notifications pre- and during COVID-19 was conducted (cut-off date: COVID-19 emergency declaration in Peru, 16 March 2020). The effect of TB care occurring pre-vs. during COVID-19 on TB treatment success in the incarcerated and non-incarcerated populations was explored using logistic regression.

**Findings:**

DS-TB cases notified in prisons from Jan 2018 to Dec 2021 (n = 10,134) represented 10% of all cases notified in the country (n = 101,507). In the first week of COVID-19, DS-TB case notifications dropped by 61.2% (95% CI: 59.9–62.7%) in the non-incarcerated population and 17.7% (95% CI: 17.5–17.9%) among the incarcerated population. TB treatment success was significantly lower in people receiving TB care entirely during the COVID-19 pandemic vs. before COVID-19 in the non-incarcerated population (OR: 0.81, 95% CI: 0.78–0.85), but not statistically significantly lower in the incarcerated population (OR: 0.88, 95% CI: 0.76–1.01). Incarceration status was not found to modify the effect of COVID-19 period on TB treatment outcomes (OR: 1.07, 95% CI: 0.92–1.25), although treatment success was higher in the incarcerated population (OR [incarcerated vs. not incarcerated, pre-COVID]: 1.52, 95% CI: 1.39–1.67).

**Interpretation:**

Both incarcerated and non-incarcerated populations experienced a large drop in DS-TB case notifications (although higher in the non-incarcerated population). Lower TB treatment success among those receiving care during COVID-19 indicates significant TB service disruptions in the overall population. The finding that incarceration at time of diagnosis was associated with treatment success is plausible in Peru given increased screening and stricter treatment monitoring in prisons.

**Funding:**

Canadian Institutes of Health Research (Funding Reference Number: 179418) .

**Research in context**

**Evidence before this study**

An initial literature search on TB treatment outcomes in prisons, using the Embase and Medline databases (via Ovid) was conducted in September 2022, and updated in August 2023, including studies published from the year 2000 to the search date (August 2023 for the updated search), and used the search terms (prison∗ OR jail OR incarcerat∗ OR detain∗ OR detention OR deprived of liberty) AND (tuberculosis) AND (treatment outcome∗ OR treatment completion OR treatment success). This search identified 211 studies, of which 27 compared TB outcomes among incarcerated vs. non-incarcerated populations. These studies were conducted in the USA (n = 4), Brazil (n = 3), Iran (n = 3), the Netherlands (n = 2), Georgia (n = 2), Malawi, Spain, Portugal, Estonia, Latvia, Moldova, Kyrgyzstan, Thailand, Russia, Kazakhstan, and Australia (n = 1 in each country), or across multiple countries (n = 2). Studies reported heterogeneous findings regarding treatment success, with treatment success being lower in incarcerated populations in some settings due to risk factors and barriers to care being prevalent in the carceral setting, whilst in other settings, more favourable treatment outcomes have been observed among people in prisons due to stricter treatment monitoring and more frequent screening. This highlights that TB outcomes in prisons are context-dependent. In addition, although it is also known that the COVID-19 pandemic significantly disrupted TB programs globally, none of these studies evaluated the impact of COVID-19 on TB notifications and treatment outcomes in incarcerated and non-incarcerated populations. Lastly, restricting the above search to studies conducted in Peru, but removing the search terms related to treatment outcomes (to return any studies on TB in prisons in Peru, regardless of study objective) identified one study including participants from Peru and four other countries that found history of incarceration to be associated with loss to follow-up during TB treatment. However, results were not stratified by country and Peru-specific estimates are therefore not available. Two further studies conducted in Peru investigated the role of prisons in TB transmission to the general community, and two others surveyed detainees to assess the prevalence of TB in prisons in Peru, finding a high prevalence, however, these estimates are limited by reliance on self-reported TB diagnoses. This highlights that TB in prisons in Peru remains under-studied, despite overcrowding in prisons in the country being an urgent and growing concern for infectious disease prevention.

**Added value of this study**

This national-level study of DS-TB case notifications and treatment outcomes in the incarcerated and non-incarcerated populations of Peru contributes to our understanding of disruptions to TB services during the pandemic in these populations. We found extremely high DS-TB notification rates in prisons in Peru, highlighting incarcerated populations as a key population for TB elimination in Peru. In addition, we found a higher drop in case notifications in the non-incarcerated population than in the incarcerated population in the first week of the COVID-19 pandemic. TB treatment outcomes were also affected by COVID-19-related disruptions, with TB treatment success in the non-incarcerated population being significantly lower among those receiving all of their TB care during the pandemic (compared to before the pandemic). Our finding that the likelihood of treatment success was higher among people receiving their TB diagnosis in prison (possibly due to increased screening and treatment monitoring in prisons in Peru) contributes to existing knowledge on TB treatment outcomes in prisons, as findings from studies in other countries have been heterogeneous, underlining the importance of country-specific studies, given that carceral settings vary across countries. Therefore, this study adds to existing literature on TB in incarcerated populations, particularly in Peru, where TB in prisons is under-studied.

**Implications of all the available evidence**

We highlight that the burden of TB in prisons in Peru is extremely high compared to the general population in the country. The drop in case notifications during COVID-19 in both the incarcerated and non-incarcerated populations suggests a high number of missed cases, potentially hindering early treatment initiation and favourable outcomes among patients. The finding that TB treatment success was significantly lower in those receiving TB care during COVID-19 (in the non-incarcerated population) highlights significant setbacks in TB care incurred during the pandemic. The resilience of TB services in the carceral setting to external disruptions such as those seen during the COVID-19 pandemic, in combination with the high burden of TB in this population, highlights prisons as unique settings in which TB prevention and treatment is feasible yet urgent, making clear that prisons should be key priorities for TB elimination efforts. Further studies on TB among people in prison are needed, particularly those using a dynamic indicator of incarceration status (which was not possible in this study).

## Introduction

The COVID-19 pandemic has set back progress on ending TB globally, with the World Health Organisation (WHO) reporting increases in TB incidence and mortality in recent years.^1^, ^2^, ^3^ The COVID-19 pandemic has severely disrupted TB control in Peru,^4^^,^^5^ which suffered the 13th largest drop in TB case notifications from 2019 to 2020 of all countries worldwide.^3^ In light of these disruptions, it is urgent to focus TB elimination efforts on key populations, such as people experiencing incarceration, who are at particularly high risk for TB. We therefore evaluate TB case notifications and treatment outcomes among the incarcerated and non-incarcerated populations in Peru, before compared to during the COVID-19 pandemic.

Studies in a variety of countries report an extremely high prevalence and incidence of TB in incarcerated populations.^6^, ^7^, ^8^, ^9^, ^10^, ^11^, ^12^ Prisons in South America, however, have been found to have a particularly high burden of TB, with a systematic review estimating a pooled TB prevalence of 1680 cases/100,000 persons (95% CI: 830–2970) in the region.^12^ In Peru, results of a 2016 survey of detainees found a prevalence of 2510 cases/100,000 persons of self-reported TB diagnosis (detainees reported having been diagnosed by a healthcare provider) during incarceration.^13^

Findings regarding TB treatment outcomes in prisons in previous studies are variable. Barriers to successful TB treatment may be exacerbated in the prison setting by high rates of tobacco, drug and alcohol use,^14^ and some studies report low proportions of treatment success.^15^^,^^16^ Other studies, however, have found higher treatment success among people living in prisons than among those not incarcerated, including a large study in Brazil (n = 17,776 incarcerated, n = 160,728 non-incarcerated) that found higher treatment success in the incarcerated population (82.2% vs. 75.1%, p < 0.0001) due to more widespread implementation of DOTs in the prison setting.^17^

Regarding the impact of the COVID-19 pandemic in places of detention, COVID-19 cases and deaths are much higher in prisons compared to the general population.^18^, ^19^, ^20^ Prison settings also experienced larger COVID-19-related disruptions as prisons were de-prioritized in the pandemic response (including in COVID-19 vaccine roll-out).^21^^,^^22^ In addition, limited health resources in prisons have been diverted towards the pandemic response,^23^^,^^24^ resulting in restrictions of detainees’ medical appointments outside of prison, long delays in care pathways,^23^ and, in some cases, the limitation of prison health services to emergency-only levels.^22^ The pandemic also aggravated already-dire staff shortages in prisons,^22^ and although some settings enacted decarceration measures to alleviate pressure on prison health systems, these were temporary.^21^^,^^25^, ^26^, ^27^

There are currently no studies on TB treatment outcomes in incarcerated and non-incarcerated populations in Peru. In addition, the COVID-19 pandemic has now presented further challenges for TB care in both the general population and in prisons. Although TB control globally has been severely disrupted by COVID-19,^1^ specific high-risk settings, such as prisons, have not been evaluated despite widespread reports of disrupted health services in prisons.^10^^,^^11^ This study therefore aimed to describe DS-TB case notifications in the overall and incarcerated population in each department (largest administrative division) of Peru, assess trends in weekly national DS-TB case notifications prior to vs. during COVID-19, and identify predictors of treatment success (treatment completion or cure) in incarcerated and non-incarcerated people in Peru, prior to vs. during the COVID-19 pandemic. To assess trends in the DS-TB case notifications prior to and during the pandemic, an interrupted time series (ITS) approach is used. This allows extrapolation of the pre-COVID-19 trend in the during-COVID-19 period (counterfactual) and comparison of this to the observed during-COVID-19 trend.^28^

## Methods

### Study setting and context

#### COVID-19 in Peru

**Fig. 1:**
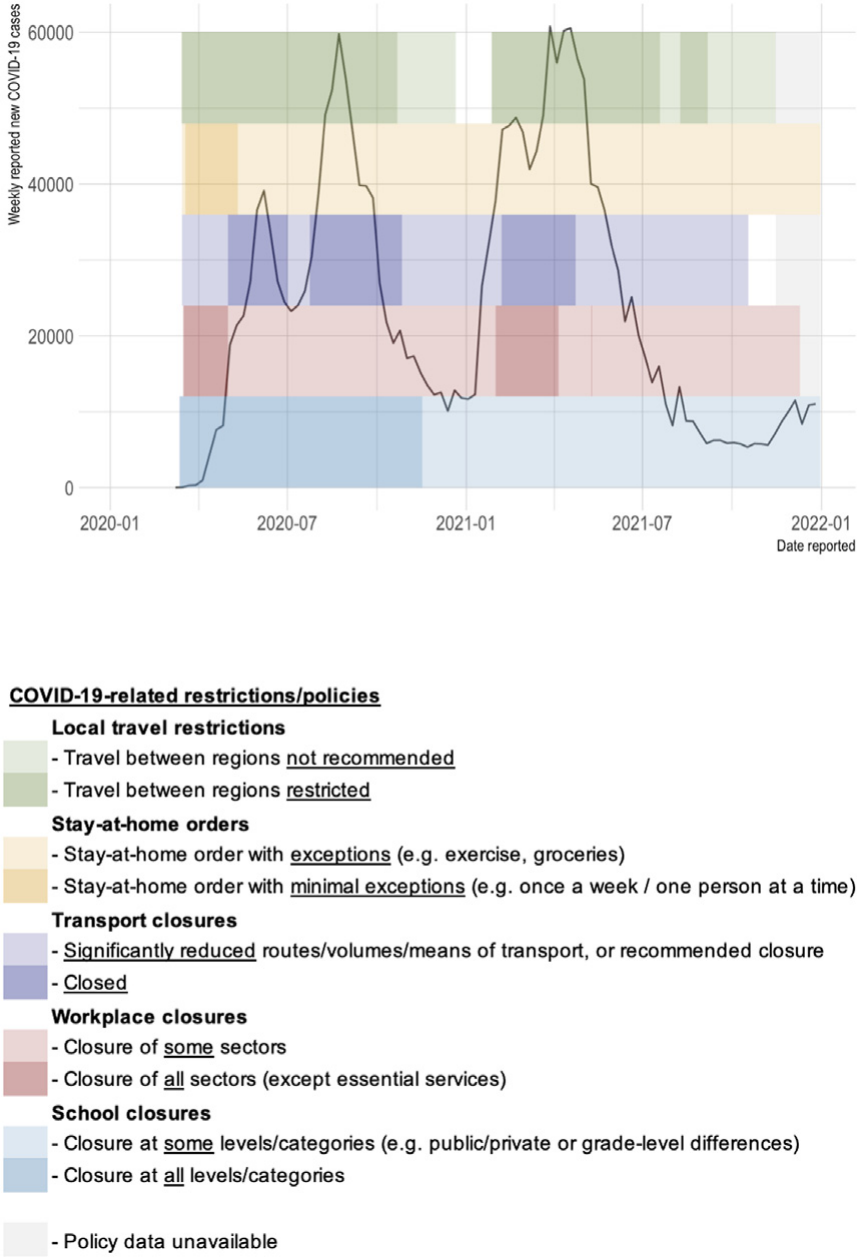
Weekly new COVID-19 cases reported in Peru,^29^ and COVID-19-related restrictions coinciding with the study period (Jan 2020–Dec 2021).^30^ COVID-19 cases are from the WHO COVID-19 database.^29^ COVID-19 measures/policies are from the Oxford COVID-19 Government Response Tracker.^30^

For context on disruptions during the COVID-19 pandemic in Peru, COVID-19 case surges as well as the associated restrictions (closures of schools, workplaces, transport, etc.) are shown in Fig. 1 (COVID-19 cases from the WHO COVID-19 database,^29^ and COVID-19 measures/policies from the Oxford COVID-19 Government Response Tracker^30^).

#### Incarceration in Peru

As of September 2023, over 93,000 people are living in prisons in Peru,^31^ (incarceration rate: 273/100,000 people^32^). Supplementary Fig. S1 shows trends in the incarcerated population size over the study period (2018–2021). On average, prisons in the country are at 220% capacity,^31^ creating aforementioned risks for increased transmission of TB, COVID-19, and other infectious diseases.^33^ In response to the COVID-19 pandemic, approximately 7,900 detainees have been released since 2020 under the decriminalization laws enacted to reduce the incarcerated population.^34^

#### TB management in prisons in Peru

The following information on TB management in prisons in Peru is based on observations and discussions with penitentiary health staff during visits (reported here by the author and collaborators) to two large prison facilities in Peru. It should therefore be noted that specifics pertaining to the availability of TB services may vary by facility, particularly in smaller or more remote prisons.

Depending on their size, prison establishments in Peru have dedicated TB care facilities and staff. Detainees receive a physical health evaluation and a chest X-ray at entry, and those with abnormal chest X-ray findings undergo sputum microscopy. GeneXpert platforms for TB testing, provided by the Global Fund, are available in 3 of the country’s 68 prisons. Large prisons may have laboratories for sputum microscopy on-site, although samples are sent to external reference laboratories for culture and drug-susceptibility testing.

Detained persons diagnosed with TB stay in cell blocks separate from healthy detainees (at least until smear negative) and receive additional food (4 meals per day). DOT is provided either directly in the cells, or at a TB treatment area within the prison. Treatment for extensively drug-resistant (XDR) TB is centralised at one specific prison in the country. If a detainee is released during TB treatment, they are referred to their local primary care centre for continuation of treatment, although in practice ensuring continuity of care in this context is difficult. Although reduced staff and the attribution of respiratory symptoms to COVID-19 complicated TB care in prisons during the COVID-19 pandemic (likely reducing case detection), prison clinic hours, DOTS and treatment monitoring continued as normal, according to discussions with penitentiary health staff.

### Study population

We included all adults (aged ≥ 18 years) diagnosed with drug-susceptible TB (pulmonary and extrapulmonary) from 1 Jan 2018 to 31 Dec 2021 (refer to section below for analysis-specific timeframes within this period) and reported in the Peruvian national TB program database, Sistema de Información Gerencial de Tuberculosis (SIGTB).^35^

### Data source

SIGTB^35^ includes information on all notified TB cases in Peru, including TB diagnoses within the prison system, as well as basic demographic and clinical information (age, sex, HIV status, diabetes status, type of TB (pulmonary/extra-pulmonary), drug-resistance status, treatment outcome).

For the purpose of this study, TB among incarcerated individuals is defined as cases diagnosed during incarceration, with those receiving a TB diagnosis after release from a prior incarceration not being included in this category. This is because incarceration status is only available in SIGTB for those receiving a TB diagnosis during incarceration (prior incarceration status and length of incarceration is not captured).

### Timeframes and COVID-19 period definitions

Definitions of pre-vs. during COVID-19 periods for each analysis are described below.

#### Department-level DS-TB case notifications in the incarcerated & non-incarcerated populations pre- & during COVID-19

Case notifications in the one-year timeframe prior to COVID-19 (based on date of diagnosis as recorded in the NTP database) were compared to notifications in the first year of the COVID-19 pandemic. This analysis therefore includes those diagnosed between 15 March 2019 and 15 March 2020 (pre-COVID-19) and those diagnosed between 16 March 2020 and 16 March 2021 (during COVID-19).

#### Interrupted time series analysis of DS-TB case notifications pre- & during COVID-19

This analysis includes all cases notified between 1 Jan 2018 and 31 Dec 2021. As pandemic restrictions and the declaration of a national state of emergency came into effect in Peru on 16 March 2020,^36^ the week of 16 March 2020 is defined as the first week of COVID-19 in the ITS analysis.

#### DS-TB treatment outcomes in the incarcerated & non-incarcerated populations pre- & during COVID-19

This analysis includes those diagnosed between 1 Jan 2018 and 16 Mar 2021. As this analysis assesses TB treatment outcomes rather than case notifications, treatment time is taken into account in the categorization of COVID-19 periods into three categories: TB care occurring pre-COVID-19, partially during COVID-19, and fully during COVID-19. For example, those on a 6-month standard regimen for DS-TB were defined as having received TB care prior to COVID-19 if they were diagnosed between 1 Jan 2018 and 15 Sep 2019, meaning they would have been expected to complete their 6-month treatment prior to the start of COVID-19 restrictions on 16 Mar 2020. Similarly, those on a 6-month regimen diagnosed between 16 Sep 2019 and 15 Mar 2020 were defined as having received their TB care partially during COVID-19, and those diagnosed 16 Mar 2020 to 16 Mar 2021 were defined as receiving their TB care fully during COVID-19. For detail on COVID-19 period definitions adapted to those receiving longer regimens (9-month and 12-month),^37^ please refer to Table 1.

**Table 1:** COVID-19 period definitions for assessing tuberculosis treatment outcomes prior to and during COVID-19 in Peru.

TB treatment regimen	Regimen treatment time	TB care pre-COVID-19	TB care partially during COVID-19	TB care fully during COVID-19
2HREZ/4(HR)3^a^	6 months	Diagnosed 1 Jan 2018–15 Sep 2019 (≥6 months prior to start of COVID-19 restrictions)	Diagnosed 16 Sep 2019–15 Mar 2020	Diagnosed 16 Mar 2020–16 Mar 2021
2HREZ/7HR^b^	9 months	Diagnosed 1 Jan 2018–15 June 2019 (≥9 months prior to start of COVID-19 restrictions)	Diagnosed 16 June 2019–15 Mar 2020	
2HREZ/10HR^c^	12 months	Diagnosed 1 Jan 2018–15 Mar 2019 (≥12 months prior to start of COVID-19 restrictions)	Diagnosed 16 Mar 2019–15 Mar 2020	

H = isoniazid, R = rifampicin, E = ethambutol, Z = pyrazinamide.

a Daily∗ HREZ for 2 months, HR for 4 months on 3 days per week (Standard DS-TB regimen). ∗Except Sundays.^37^

b Daily∗ HREZ for 2 months, daily∗ HR for 7 months (DS-TB regimen in people living with HIV). ∗Except Sundays.^37^

c Daily∗ HREZ for 2 months, daily∗ HR for 10 months (Extrapulmonary DS-TB regimen). ∗Except Sundays.^37^

### Statistical analysis

R (version 4.1.2) was used for all statistical analyses.^38^

#### Department-level DS-TB case notifications in the incarcerated & non-incarcerated populations pre- & during COVID-19

Departments are the largest administrative division of Peru (followed by provinces and districts), and there are 25 departments in the country in total (24 plus the constitutional province of Callao).^39^ TB case notifications in the incarcerated population were compared with the general population in each department by calculating incidence rate ratios (IRR) (Supplementary Section 1).^12^ Department-level IRRs are also presented for the pre-COVID-19 time period (diagnoses between 15 March 2019 and 15 March 2020) and during the COVID-19 pandemic (diagnoses between 16 March 2020 and 16 March 2021).

#### Interrupted time series analysis of DS-TB case notifications pre- & during COVID-19

An interrupted time series was conducted for weekly DS-TB case notifications in SIGTB from 1 Jan 2018 to 31 Dec 2021 (209 weeks in total). The start of the COVID-19 pandemic was considered the date of the declaration of the national emergency due to COVID-19, 16 Mar 2020 (week 116 of 209). As this was a Monday, weeks in the time series were defined as starting on Mondays.

To model weekly case counts, a negative binomial model was used to account for overdispersion of the data. We present observed case notifications and counterfactual trends expected in the absence of the COVID-19 pandemic.^40^ Three separate models were fit, for case notifications among 1) the total population of Peru 2) the non-incarcerated population, and 3) the incarcerated population.

Based on the observed temporal trend in case notification in the total population, three time periods were defined: Phase 1, the pre-COVID-19 phase (weeks 1–115, i.e., 1 Jan 2018 to 15 Mar 2020), phase 2–the recovery phase (weeks 116–151, i.e., 16 Mar 2020 to 22 Nov 2020), and phase 3–the post-recovery phase (weeks 152–209, i.e., 23 Nov 2020 to 31 Dec 2021). In phase 2, cases increased linearly after the initial drop due to COVID-19, and in phase 3 the temporal trend began to resemble phase 1. Seasonality trends were modelled using harmonic terms. In the simplest case, the same harmonic term was used for the duration of the time series. However, if the seasonality pattern changed across phases, distinct harmonic terms were used for each phase. Dichotomous indicators were used to modify the intercept, slope, and harmonic terms for the respective phases (Supplementary Figs. S2–S4).

All models included the log of the population size as an offset term, to account for fluctuations in the source population. In particular, notable changes in the incarcerated population occurred due to decarceration efforts during the COVID-19 pandemic (Supplementary Fig. S1). Estimates of the total population size were based on annual population projections from the Peruvian census,^41^ the population size for those incarcerated was based on monthly counts of the incarcerated population from Peru’s penitentiary institute database,^42^ and the non-incarcerated population size was based on annual population estimates from the national census, minus the average annual incarcerated population. Model equations are shown in the Supplementary Equations S1–S3.

Counterfactual values for expected case notifications in the absence of COVID-19 were estimated using the Phase 1 (pre-COVID-19) model parameters. As the incarcerated population decreased in the COVID-19 pandemic period (refer to Supplementary Fig. S1) as a result of COVID-19-related decarceration policies, the monthly incarcerated population sizes used in the counterfactual scenario were incrementally increased by n = 388, the average monthly change in the incarcerated population size in the year prior to the COVID-19 pandemic (2019).

#### DS-TB treatment outcomes in the incarcerated & non-incarcerated populations pre- & during COVID-19

The impact of receiving TB care prior to, partially during or fully during the COVID-19 pandemic (according to definitions in Table 1) on TB treatment success in the incarcerated and non-incarcerated populations was explored using logistic regression (refer to Supplementary Equation S4). Relevant covariates adjusted for included sex, age, HIV status, diabetes status, smoking, extrapulmonary vs. pulmonary TB, new vs. relapse case, TB treatment adherence, and health insurance type. The non-linear relationship between age and likelihood of treatment success was modelled flexibly using penalised splines. Separate models were fit for 1) the total population, 2) the non-incarcerated population, and 3) the incarcerated population. In an additional analysis among the total population, the interaction between COVID-19 period and incarceration status was also explored.

Lacking other indicators of socioeconomic status in the national database, health insurance type was included as a proxy for socioeconomic status. There are various categories of health insurance in Peru; Seguro Integral de Salud (SIS), Seguro Social de Salud (EsSalud), and private insurance.^4^ SIS is Peru’s free public health insurance, serving the lower-income population, while EsSalud is insurance provided to workers via their employer (note: individuals employed in the police or armed forces have separate health insurance financed by the Ministry of Defence.^4^ This is included in the worker’s insurance category for the purpose of our analysis). Lastly, for additional benefits, private insurance can also be obtained by those able to pay higher premiums. Health insurance type is therefore expected to be a relevant (although imperfect) proxy for socioeconomic status.

As only the number of doses at exit (end of follow-up, completion, or death, etc) were available in the national TB program database, adherence is estimated based on the number of doses at exit, vs. total doses expected for the individual’s treatment regimen and their time on treatment before exit. Implausibly high values for doses at exit were recoded as missing.

TB treatment success was defined as either cure or treatment completion. Treatment drop-out, treatment failure, the development of drug-resistant TB, or death were classified as unsuccessful treatment outcomes. The development of drug-resistance or other reasons for permanent treatment suspensions or changes were also classified as failures of first-line treatment, as per updated WHO treatment outcome definitions.^43^

Covariates with missing data included diabetes (8.2% missing), HIV (7.3% missing), adherence (2.6% missing), health insurance type (0.2% missing), smoking (0.2% missing), site (pulmonary/extrapulmonary) of TB (0.01% missing), and COVID-19 period (0.004% missing–due to missingness of treatment regimen, pre/during COVID-19 TB treatment was not assigned in these cases, given this was defined based on expected treatment length). Missing data on covariates were imputed by multiple imputation by chained equations (MICE) (using the ‘mice’ package in R),^44^ and the imputed data used in final analyses. Individuals with missing outcome data (2.9%) were excluded from analyses. Sensitivity analyses were conducted to assess robustness of results, including the effect on model estimates of the use of MICE for imputation of missing covariate data compared to complete case analysis, and classifying those with missing outcome data as having unsuccessful outcomes.

#### Power calculation

Our sample size was fixed by design, as it included all adult TB notifications in Peru in the study period.

### Research ethics

Ethics approval was obtained by McGill University (McGill IRB: A09-M51-21A) and Universidad Peruana Cayetano Heredia (UPCH IRB SIDISI: 209660). Approval to use SIGTB data was granted by the Peruvian National TB program. The study protocol was also presented to representatives of the Peruvian National Penitentiary Institute. This study is registered in the Peruvian National Institute of Health’s study registry (PRISA) (EI00002907).

### Role of the funding source

LF was supported by a Canadian Institutes of Health Research Doctoral Award and subsequently by a Banting Postdoctoral Fellowship. MP was supported by a Canada Research Chair award from the Canadian Institutes of Health Research. SH was supported by a Canadian Institute of Health Research Postdoctoral Fellowship. Listed funding represents career or educational support only; the funders did not have any role in the conceptualization or writing of the manuscript or in the decision to submit it for publication.

## Results

### DS-TB case notifications in the incarcerated and non-incarcerated populations pre- and during COVID-19, by region (department)

**Fig. 2:**
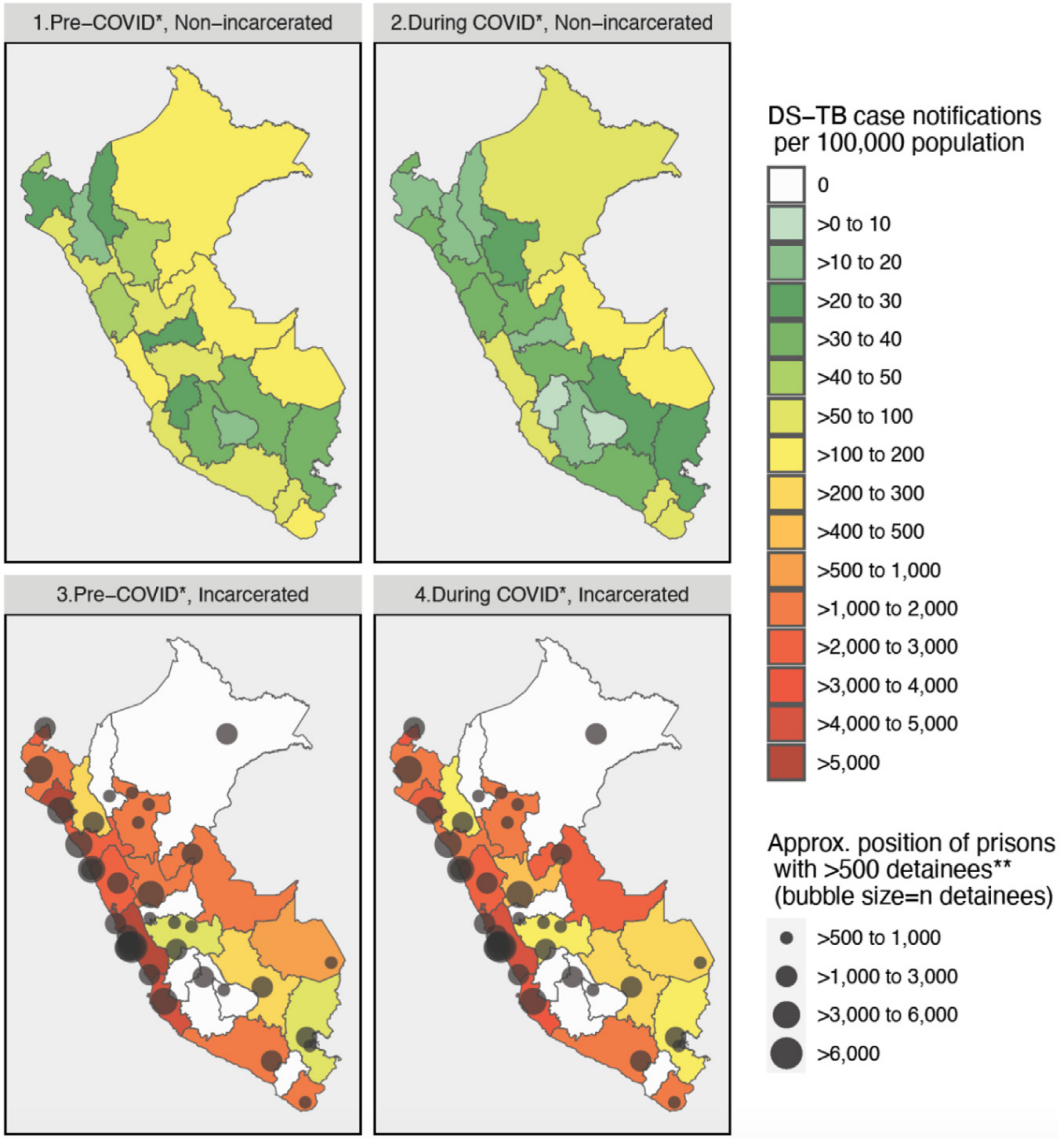
Title: Drug-susceptible tuberculosis case notifications in the incarcerated vs. non-incarcerated population, per 100,000 people, Pre-and during COVID-19, in Peru, by department (March 2019 to March 2021). Denominators for incidence in incarcerated and non-incarcerated populations: Pre-COVID-19 incarcerated: average incarcerated population of department, Mar 2019 to Feb 2020 (from publicly available INPE data).^42^ During COVID-19 incarcerated: average incarcerated population of department, Mar 2020–Feb 2021 (from publicly available INPE data).^42^ Pre-COVID-19 non-incarcerated: census projection for overall 2019 department population,^39^ minus department’s pre-COVID prisoner population above. During COVID-19 non-incarcerated: census projection for overall 2020 department population,^39^ minus department’s during-COVID prisoner population above. ∗Pre-COVID-19 = DS-TB diagnosis between 15 March 2019 and 15 March 2020. During COVID-19 = DS-TB diagnosis between 16 March 2020 (start of COVID-19 restrictions in Peru) and 16 March 2021. ∗∗Showing only prisons with >500 detainees as of March 2019 (n = 37 out of a total of 69 prisons in the country at the time).^42^

This analysis was based on case notifications in the year prior to COVID-19 (15 Mar 2019–15 Mar 2020) and the first year of COVID-19 (16 Mar 2020–16 Mar 2021) (analysis-specific timeframes described in Methods). Between 15 Mar 2019 and 16 Mar 2021, 48,352 adult DS-TB cases were notified in Peru, of which 4776 (9.9%) were incarcerated at the time of their TB diagnosis. Department-level case notifications per 100,000 people are shown in Fig. 2, by incarceration status and COVID-19 period, and including approximate locations of the country’s largest prisons. Exact notification rates by department, and for the total population of Peru, are shown in Supplementary Table S1. The country-level notification rates (for all of Peru) in the total population (incarcerated and non-incarcerated) were 89 DS-TB cases per 100,000 people in the year prior to COVID-19 and 60 DS-TB cases per 100,000 in the first year of the pandemic. In the non-incarcerated population, the rates were 81 and 54 DS-TB cases per 100,000 people in the pre-vs. during COVID-19 periods, respectively. In prisons in Peru, notification rates were extremely high, with 2894 DS-TB cases per 100,000 people notified in the year prior to COVID-19, and 2245 cases per 100,000 people in the first year of COVID-19.

By department, notification rates in the overall population prior to the COVID-19 pandemic ranged from 15 cases per 100,000 people (in Cajamarca) to 182 per 100,000 people (in Ucayali) and ranged from 9 per 100,000 (in Huancavelica) to 119 per 100,000 people (in Ucayali) in the first year of the pandemic. In the incarcerated population, notifications rates by department (for departments reporting at least one TB case in prisons) ranged from 84 per 100,000 (in Puno) to 8332 per 100,000 (in Lambayeque) prior to the pandemic, and from 107 per 100,000 (in Cajamarca) to 4143 per 100,000 (in Lima) during the pandemic.

**Fig. 3:**
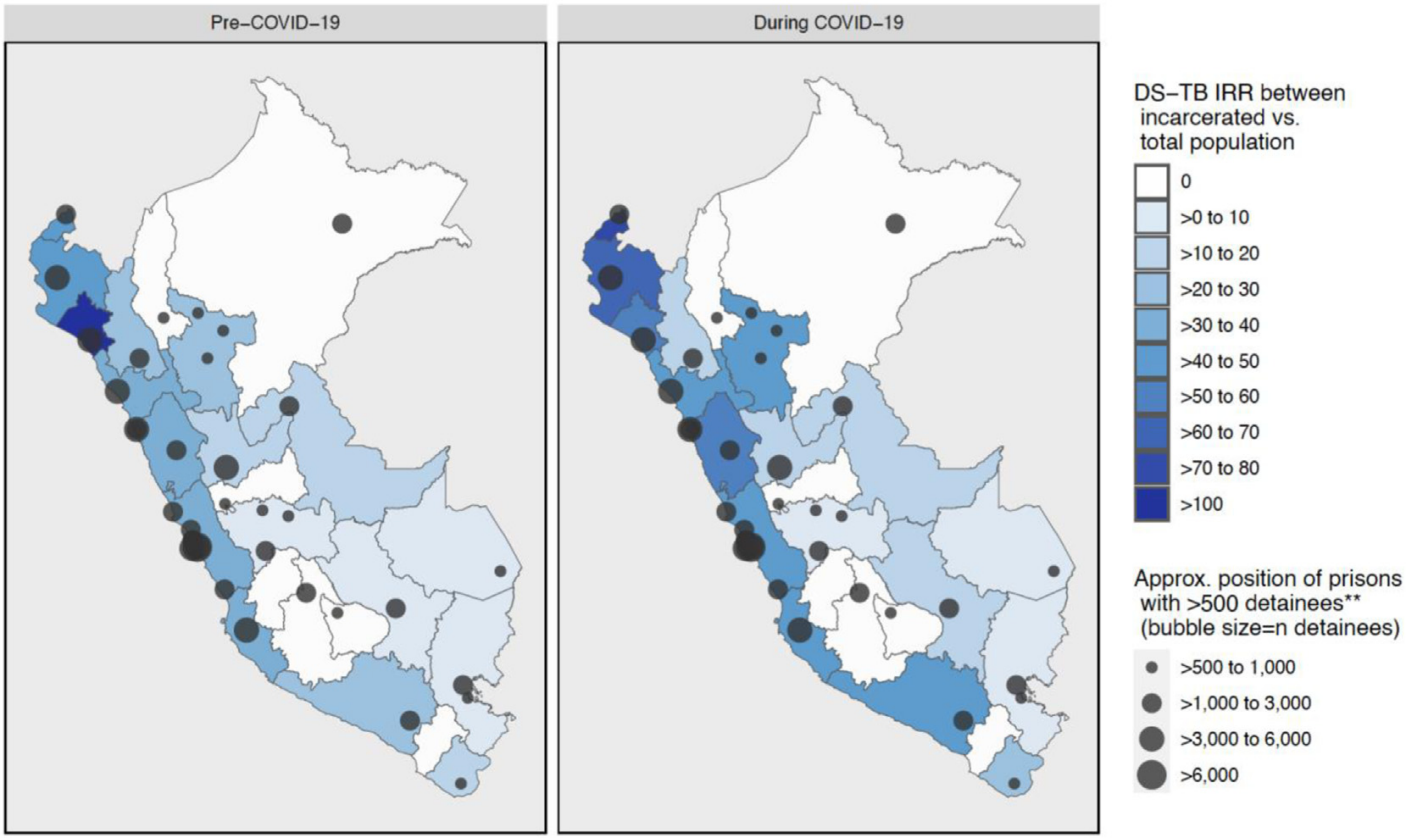
Drug-susceptible tuberculosis incidence rate ratio between the incarcerated and total population pre- and during COVID-19∗ in Peru, by department (March 2019–March 2021). IRR = Incidence rate ratio = DS-TB case notifications per 100,000 in prisons/overall DS-TB case notifications per 100,000 in department. Denominators for incidence in incarcerated and total population: Pre-COVID-19, incarcerated: average incarcerated population of department, Mar 2019 to Feb 2020 (from publicly available INPE data).^42^ During COVID-19, incarcerated: average incarcerated population of department, Mar 2020 to Feb 2021 (from publicly available INPE data).^42^ Pre-COVID-19, total population: census projection for overall 2019 department population.^39^ During COVID-19, total population: census projection for overall 2020 department population.^39^ ∗Pre-COVID-19 = DS-TB diagnosis between 15 March 2019 and 15 March 2020. During COVID-19 = DS-TB diagnosis between 16 March 2020 (start of COVID-19 restrictions in Peru) and 16 March 2021. ∗∗Showing only prisons with >500 detainees as of March 2019 (n = 37 out of a total of 69 prisons in the country at the time).^42^

To compare case notifications in the prison population to the overall population of each department, incidence rate ratios (IRRs) are presented in Fig. 3. It should be noted that IRRs here represent notifications in prisons in each department divided by overall notifications in the department, and notifications do not necessarily represent true incidence given under-diagnosis and under-notification of incident TB cases. In addition, it should be noted that changes in IRRs can reflect changes in both the numerator and denominator. Exact IRRs by department are listed in Supplementary Table S1. IRRs between prisons and the total population are high, echoing previous findings from South America.^12^ The IRR between the incarcerated and total population of Peru was 32.37 in the year prior to COVID-19, and 37.32 in the first year of COVID-19. Overall, IRRs during COVID-19 increased compared to pre-COVID-19 IRRs, due to the larger drop in notifications in the general population compared to in the incarcerated population (covered in more detail in the time series analysis).

### Interrupted time series analysis of DS-TB case notifications pre- and during COVID-19

**Fig. 4:**
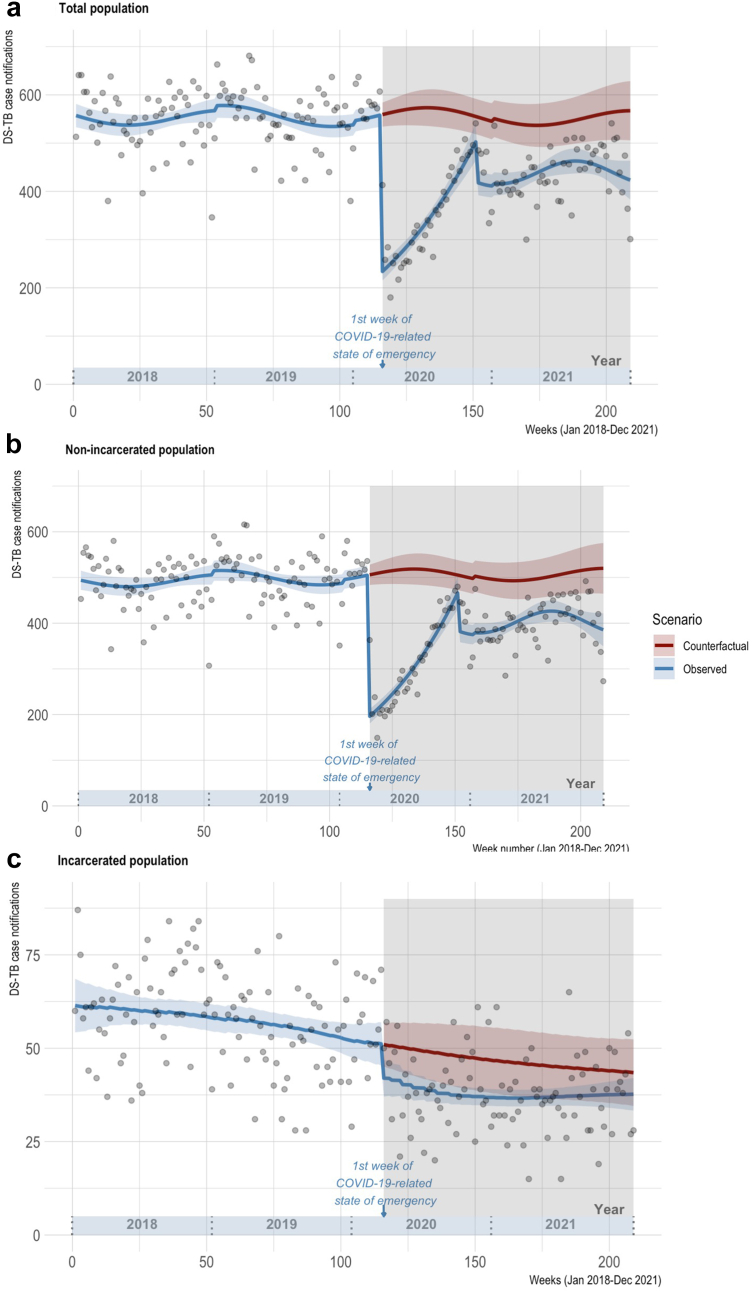
Interrupted time series of weekly DS-TB case notifications in Peru pre- and during COVID-19, 2018–2021. Red & blue shaded areas = 95% CIs. Points = actual weekly case counts. Gray shaded area = COVID-19 pandemic period (following COVID-19-related national state of emergency declaration in Peru on 16 March 2020).^36^

From 1 Jan 2018 to 31 Dec 2021, 101,507 adult DS-TB cases were notified in the national TB program database, of which 10,134 (10.0%) were notified in individuals who were incarcerated at the time of their TB diagnosis. Observed weekly case notifications and the counterfactual trend of notifications expected in the absence of the COVID-19 pandemic in the total, non-incarcerated, and incarcerated populations are shown in Fig. 4. Case notifications as rates per 100,000 population in the incarcerated and non-incarcerated populations are provided in Supplementary Fig. S4.

Case notifications among the total Peruvian population (Fig. 4a) dropped sharply in the first week of the enactment of COVID-19-related restrictions in Peru (week 116) compared to the counterfactual expected number of cases in the same week, with 234 predicted cases (95% CI: 215–253) (in terms of rates, 0.72 cases per 100,000 people, 95% CI: 0.66–0.78) in the observed scenario (start of COVID-19) compared to 559 cases (95% CI: 534–584) (1.71 cases per 100,000 people, 95% CI: 1.64–1.79) in the counterfactual scenario (absence of COVID-19). This represents a 58.1% drop in cases (95% CI: 56.7–59.7) in the observed vs. counterfactual scenarios (Table 2).

**Table 2:** Observed and counterfactual weekly DS-TB case notifications^a^ in the first week of the COVID-19 emergency declaration in Peru.

Scenario	Total population			Non-incarcerated population			Incarcerated population		
	Cases	95% CI		Cases	95% CI		Cases	95% CI	
Observed	234	215	253	196	180	212	42	37	47
Counterfactual	559	534	584	506	484	528	51	45	57

	% Drop^b^	95% CI		% Drop^b^	95% CI		% Drop^b^	95% CI	
Observed vs. Counterfactual	58.1	56.7	59.7	61.2	59.9	62.7	17.7	17.5	17.9

a Based on predicted values from each model.

b % drop = $\frac{counterfactual\;cases\;-\;observed\;cases}{counterfactual\;cases}$ ∗ 100.

Upon stratifying non-incarcerated and incarcerated populations, it becomes clear that the overall drop in case notifications was greater among the non-incarcerated population. Among the non-incarcerated population (Figs. 4b), 196 cases (95% CI: 180–212) were predicted in week 116 under the observed scenario, vs. 506 cases (95% CI: 484–528) expected in the absence of COVID-19, suggesting a 61.2% (95% CI: 59.9–62.7%) drop in notifications in the first week of the pandemic. Among the incarcerated population (Fig. 4c), however, 42 cases (95% CI: 37–47) were expected in week 116 under the observed scenario, vs. 51 cases (95% CI: 45–57) in the absence of COVID-19, representing a 17.7% (95% CI: 17.5–17.9%) drop.

Following the drop in case notifications in the first week of COVID-19, case notifications gradually recovered, with the notification gap relative to the counterfactual scenario generally decreasing throughout 2020. To further describe differences in notification rates relative to the counterfactual scenario throughout the pandemic, Supplementary Table S3 shows % differences in observed vs. counterfactual DS-TB notification rates at 3-month time points. In addition, the linear components of the trends in the pre-COVID-19 and during COVID-19 phases are described in Supplementary Table S2.

### DS-TB treatment outcomes in the incarcerated and non-incarcerated populations pre- and during COVID-19

**Fig. 5:**
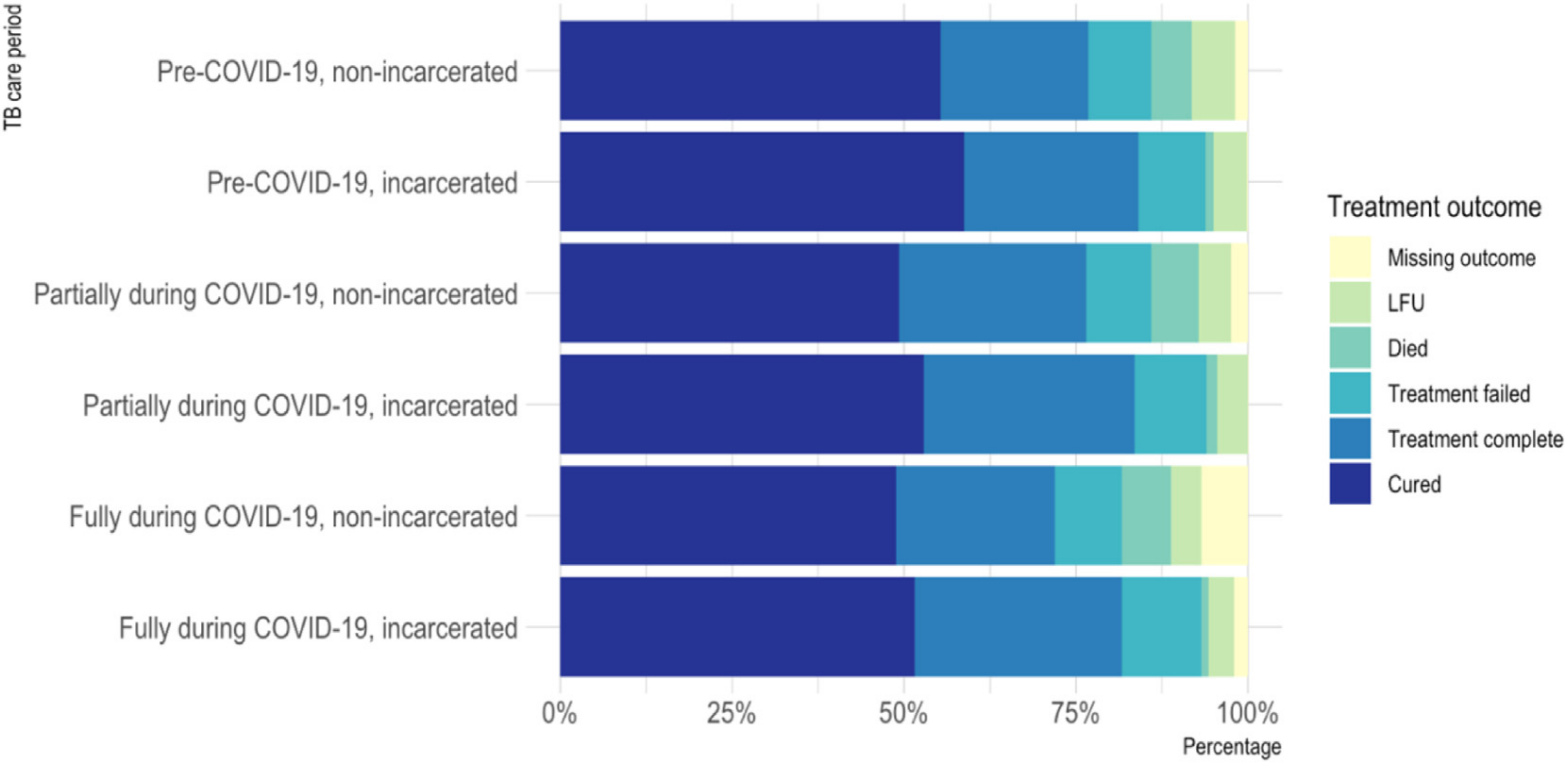
DS-TB Treatment outcomes in Peru, Jan 2018–Mar 2021, by COVID-19 period and incarceration status. TB care period: Receiving TB care pre-COVID-19, partially during, or fully during COVID-19, based on expected length of treatment time (6 m, 9 m or 12 m regimen). Refer to Table 1 for COVID-19 period explanations. LFU: lost to follow-up.

Treatment outcomes were assessed among 80,535 adults with DS-TB notified between 1 Jan 2018 and 16 Mar 2021. Of 83,039 cases notified in this period, n = 140 (0.2%) were excluded due to treatment suspension after an erroneous diagnosis of TB, and n = 2364 (2.9%) were excluded due to missing treatment outcome. The incarcerated population had a lower proportion of missing outcome data than the non-incarcerated population (0.6% vs. 3.1%, respectively) (Supplementary Table S4 and Fig. 5). The proportion of those with good adherence (≥80% of expected doses taken) was also lower in those with a missing outcome compared to those with an outcome available in the non-incarcerated population (Supplementary Table S4) (further explored in the sensitivity analysis and discussion sections).

Table 3 summarizes the characteristics of those included (n = 80,535), 8554 (10.6%) of whom were incarcerated at the time of their TB diagnosis. 47,133 individuals received their TB care pre-COVID-19, n = 15,044 partially during COVID-19, and n = 18,355 fully during COVID-19 (n = 3 missing COVID-19 grouping as missing data on treatment regimen for these 3 cases precluded COVID-19 period assignment). Those receiving their TB diagnosis during incarceration were younger than the non-incarcerated population (mean (SD): 41.2 (18.5) (non-incarcerated) vs. 32.2 (10.4) (incarcerated)), and a higher proportion were male (99.5% male (incarcerated) vs. 61.4% male (non-incarcerated)), were smokers, and had TB previously, but a lower proportion had diabetes (Table 3). The incarcerated population was also more adherent to TB treatment, likely due to stricter treatment monitoring in prisons.

**Table 3:** Characteristics^a^ of DS-TB patients in Peru by COVID-19 period and incarceration status, 1 Jan 2018–16 Mar 2021.

	Non-incarcerated			Incarcerated		
	Pre-COVID-19	Partially during COVID-19	Fully during COVID-19	Pre-COVID-19	Partially during COVID-19	Fully during COVID-19
Total	N = 41,986	N = 13,623	N = 16,369	N = 5147	N = 1421	N = 1986
Age (years)^b^	41.1 (18.8)	41.9 (18.7)	40.9 (17.7)	32.0 (10.3)	32.3 (10.5)	32.5 (10.5)
Sex (male)	25,787 (61.4%)	8457 (62.1%)	9978 (61.0%)	5122 (99.5%)	1409 (99.2%)	1977 (99.5%)
Extrapulmonary TB	8624 (20.5%)	3230 (23.7%)	2935 (17.9%)	410 (8.0%)	183 (12.9%)	227 (11.4%)
Type of case						
New	37,416 (89.1%)	12,217 (89.7%)	14,591 (89.1%)	3725 (72.4%)	951 (66.9%)	1337 (67.3%)
Previous treatment stopped or failed	1395 (3.3%)	425 (3.1%)	465 (2.8%)	74 (1.4%)	18 (1.3%)	26 (1.3%)
Relapse	3175 (7.6%)	981 (7.2%)	1313 (8.0%)	1348 (26.2%)	452 (31.8%)	623 (31.4%)
HIV positive	2117 (5.4%)	1029 (8.2%)	825 (5.6%)	139 (2.8%)	71 (5.1%)	59 (3.0%)
Diabetes	3908 (9.9%)	1317 (10.1%)	1842 (12.3%)	100 (3.4%)	49 (3.5%)	63 (3.3%)
Smoking	1896 (4.5%)	569 (4.2%)	645 (4.0%)	2532 (49.2%)	733 (51.6%)	781 (39.4%)
Type of health insurance						
Private	182 (0.4%)	51 (0.4%)	60 (0.4%)	0 (0.0%)	0 (0.0%)	0 (0.0%)
Employer's^c^	9727 (23.2%)	3188 (23.5%)	3614 (22.1%)	8 (0.2%)	2 (0.1%)	5 (0.3%)
Public^d^	25,195 (60.1%)	8762 (64.6%)	11,810 (72.4%)	5090 (98.9%)	1403 (98.9%)	1975 (99.6%)
None	6796 (16.2%)	1564 (11.5%)	836 (5.1%)	47 (0.9%)	14 (1.0%)	2 (0.1%)
TB treatment outcome						
Cured	23,651 (56.3%)	6871 (50.4%)	8577 (52.4%)	3026 (58.8%)	752 (52.9%)	1044 (52.6%)
Died	2513 (6.0%)	961 (7.1%)	1265 (7.7%)	62 (1.2%)	22 (1.5%)	21 (1.1%)
Dropped out	2681 (6.4%)	664 (4.9%)	768 (4.7%)	248 (4.8%)	62 (4.4%)	74 (3.7%)
Treatment complete	9213 (21.9%)	3804 (27.9%)	4046 (24.7%)	1309 (25.4%)	435 (30.6%)	610 (30.7%)
Treatment failed	3928 (9.4%)	1323 (9.7%)	1713 (10.5%)	502 (9.8%)	150 (10.6%)	237 (11.9%)
Good adherence (>80 of expected doses taken)	36,574 (90.0%)	12,405 (93.2%)	15,083 (94.2%)	4952 (97.8%)	1375 (98.4%)	1950 (98.9%)

a Missing data on covariates (among all, non-incarcerated, incarcerated, respectively): diabetes status (8.2%, 6.0%, 27.0%), HIV status (7.3%, 7.8%, 2.8%), adherence (2.6%, 2.8%, 1.4%), health insurance type (0.2%, 0.3%, 0.09%), smoking status (0.2%, 0.2%, 0.07%), extrapulmonary/pulmonary TB (0.01%, 0.02%, 0.0%), and COVID-19 period (0.004%, 0.004%, 0.0%).

b Age: Mean (SD). All other covariates: n (%).

c Seguro Social de Salud (EsSalud) or police/armed forces.

d Seguro Integral de Salud (SIS).

As models were fit separately for 1) non-incarcerated, 2) incarcerated, and 3) all (incarcerated and non-incarcerated) cases notified, missing data patterns were explored for all three datasets. The missing data pattern was arbitrary (non-monotone) in all three models. Missingness at random (MAR) is the likely missingness mechanism; missingness completely at random (MCAR) was ruled out (Little’s test p ≤ 0.001) (refer to Table 3 caption for breakdown of covariate missingness by incarceration status). Multiple imputation by chained equations (MICE) was used to impute missing values for covariates (using m = 20 imputations, as considered sufficient for moderately missing data).^45^ Imputation diagnostics are described in Supplementary Section S3.

**Fig. 6:**
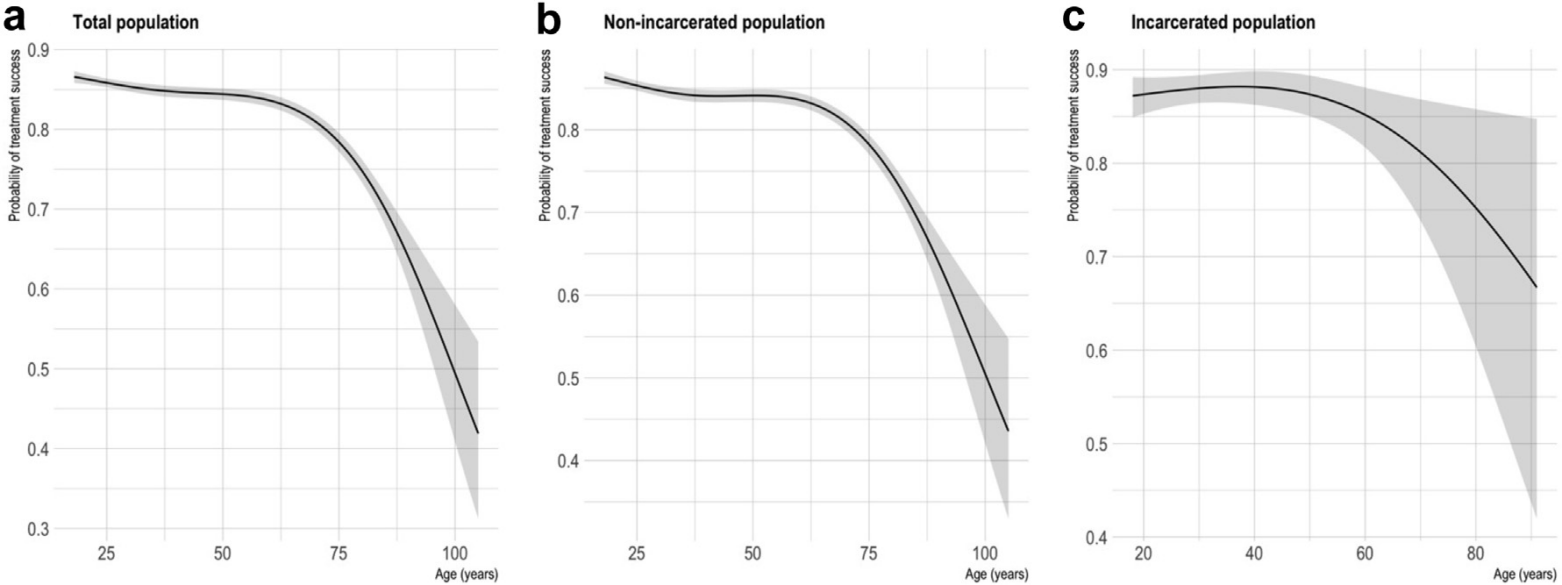
Non-linear relationship of age vs. treatment success. Penalised spline functions for age ((a) df = 4, (b) df = 4, and (c) df = 2). Plots show predicted values of probability of treatment success across values of age, holding all other covariates constant (COVID-19 period = Pre-COVID-19, sex = male, HIV = negative, diabetes = no, smoking = no, adherence category ≥ 80% of doses taken, insurance type = public, case type = new, site of TB = pulmonary) i.e., these predictions do not represent treatment success probabilities for the overall population but for a new case of pulmonary TB in a HIV-negative, non-diabetic, non-smoking, ≥ 80% adherent male with public health insurance, receiving TB care pre-COVID-19. Shaded area = 95% CI.

Results of the main analysis (using the imputed data) are shown in Table 4 (for the relationship between age and treatment success, see Fig. 6). TB treatment success was significantly lower in those receiving TB care entirely during the COVID-19 pandemic compared to prior to COVID-19 in the overall population (OR = 0.82, 95% CI: 0.78–0.85) and the non-incarcerated population (OR = 0.81 95% CI: 0.78–0.85) when adjusting for other covariates. It was also lower in the incarcerated population (although not statistically significant) (OR = 0.88, 95% CI: 0.76–1.01). The effect was less pronounced for those receiving TB care *partially* during COVID-19, suggesting less disruption in this time period (e.g., in the overall population: OR = 0.93, 95% CI: 0.89–0.98). The effect of COVID-19 on treatment success in each population on the risk scale (at fixed values of other covariates), is shown in Supplementary Table S5.

**Table 4:** Effect of COVID-19 and other covariates^a^ on DS-TB treatment success in the incarcerated and non-incarcerated population (adjusted odds ratios for treatment success from logistic regression, using imputed^b^ values for missing covariates).

	All			Non-incarcerated			Incarcerated		
	OR	95%CI		OR	95%CI		OR	95%CI	
COVID-19 period: (Ref = TB care pre-COVID-19)									
TB care fully during COVID-19	0.82	0.78	0.85	0.81	0.78	0.85	0.88	0.76	1.01
TB care partially during COVID-19	0.93	0.89	0.98	0.94	0.89	0.98	0.91	0.77	1.08
Sex, male (Ref = female)	0.81	0.77	0.84	0.79	0.76	0.82	0.93	0.40	2.16
HIV positive	0.43	0.40	0.46	0.43	0.40	0.47	0.69	0.50	0.94
Diabetes	0.98	0.92	1.04	0.99	0.93	1.06	0.74	0.52	1.04
Extrapulmonary TB	1.40	1.33	1.47	1.38	1.31	1.45	2.13	1.64	2.76
Type of case: (Ref = new case)									
Previous TB treatment stopped or failed	0.38	0.35	0.41	0.40	0.36	0.44	0.54	0.35	0.84
Relapse	0.78	0.74	0.83	0.72	0.68	0.77	0.79	0.69	0.90
Smoking	0.86	0.81	0.91	0.61	0.56	0.66	1.03	0.91	1.16
Insurance type: (Ref = private insurance)							Insurance type: (Ref = employer's insurance)^c^		
Employer's insurance^d^	0.76	0.54	1.08	0.75	0.53	1.06			
Public insurance^e^	0.67	0.48	0.95	0.64	0.45	0.90	0.83	0.19	3.71
None	0.68	0.48	0.97	0.69	0.49	0.97	1.49	0.26	8.38
Good adherence ( ≥ 80% of expected doses taken)	7.90	7.46	8.38	7.52	7.09	7.98	18.71	12.88	27.18

a For (non-linear) effect of age covariate, see Fig. 6.

b Via multiple imputation by chained equations (MICE).

c None with private insurance in incarcerated population.

d Seguro Social de Salud (EsSalud) or police/armed forces.

e Seguro Integral de Salud (SIS).

Factors that significantly reduced the likelihood of treatment success in the overall population (adjusting for other covariates) included male sex, smoking, living with HIV and relapse or previous treatment for TB. Having only public or no health insurance (compared to private health insurance), considered a proxy for socioeconomic status in our study, was also associated with lower treatment success. In the incarcerated population however, sex, smoking and health insurance type were not significant predictors of treatment outcome (Table 4).

In a further model among the total population, including an interaction term between incarceration status and COVID-19 period (Supplementary Table S6) found a higher likelihood of treatment success among people incarcerated at the time of their diagnosis (OR for treatment success among those incarcerated (vs. not incarcerated) in the pre-COVID-19 period: 1.52, 95% CI: 1.39–1.67), but also found that incarceration does not significantly modify the effect of COVID-19 on TB treatment outcome (TB care entirely during COVID-19: incarcerated, OR: 1.07, 95% CI: 0.92–1.25. TB care partially during COVID-19: incarcerated, OR: 1.03, 95% CI: 0.87–1.22).

### Sensitivity analysis

In the first sensitivity analysis, estimates using MICE to impute missing *covariate* data (the main analysis) were compared to estimates resulting from complete case analysis only (removal of observations with missing covariate data). Estimates are similar across both analyses, as shown in Supplementary Fig. S6. The second sensitivity analysis compared approaches to handling missing *outcome* data, comparing estimates from complete case analysis (deletion of those with missing outcome, the main analysis) to re-classifying those with missing outcome as having had an unsuccessful outcome—again, estimates across these approaches are similar (Supplementary Fig. S7).

## Discussion

This country-wide analysis of TB case notifications and treatment outcomes in the non-incarcerated and incarcerated populations of Peru prior to and during the COVID-19 pandemic highlights that the burden of TB is extremely high in prisons compared to the overall population, which is in line with previous studies in other South American countries^12^ but had prior to this not yet been described in Peru.

Further, although it is known that TB case notifications dropped dramatically in Peru during COVID-19,^3^ our analysis shows that this drop was much more pronounced in the non-incarcerated population than in the incarcerated population, suggesting reduced disruption of TB services in the prison setting. This is likely due to on-site TB screening at entry into prisons as well as uninterrupted identification of symptomatic patients by health promoters and TB nurses in prisons in Peru, which continued to facilitate case detection in the incarcerated population even during COVID-19. Further, fluctuations are observed in the trend in notifications in the non-incarcerated population but are absent in the incarcerated population trend. This is possibly due to TB case notifications in the incarcerated population being less sensitive to factors that may impact notifications in the general population, such as seasonality, changes in care-seeking behaviour, etc. Given the enclosed carceral setting, external factors affecting transmission may be less influential, and fluctuations in care-seeking may be less visible due to regular screening and on-site clinics in prisons.

In addition, we show that, in the non-incarcerated population, TB treatment success was significantly lower in those who received their TB care entirely or partially during the COVID-19 pandemic, compared to pre-COVID-19, but that this was not the case in the incarcerated population. This suggests COVID-19-related disruptions adversely affected TB care in Peru overall, especially in those receiving all of their TB care during the pandemic, but that disruptions experienced in the community may have differed from those experienced by incarcerated populations. For example, barriers to seeking care may have been accentuated in non-incarcerated populations who faced clinic closures or reduced clinic hours,^46^ or who had to commute to clinics in the context of transport restrictions, while incarcerated populations retained on-site access to care (as the on-site TB clinic sections of prisons remained accessible during the usual hours throughout the pandemic). Testing this via an interaction term, however, we found that incarceration is not a significant effect modifier of the relationship between COVID-19 period and TB treatment outcome; but found that incarceration (at time of diagnosis) is independently associated with a higher likelihood of treatment success. Higher TB treatment success in people experiencing incarceration has also been found in other studies,^17^^,^^47^, ^48^, ^49^ and possible explanations for this include enhanced TB screening (e.g., at entry into prison and regularly thereafter) or treatment monitoring in this population. This is plausible especially in light of specific interventions implemented to improve TB management in prisons in Peru, financed by The Global Fund,^50^^,^^51^ including early TB screening initiatives (at prison entry and annually).^51^

Similarly, the fact that the proportion of missing treatment outcome data was lower in the incarcerated than non-incarcerated population could be either due to easier on-site follow-up of patients in prisons, or due to the fact that incarcerated patients are more likely to be assigned an outcome of lost to follow-up (rather than missing) upon release from prison.

In terms of factors associated with treatment success in the overall population, our findings align with other studies that have found male sex,^52^ smoking,^53^^,^^54^ living with HIV and relapse or previous treatment for TB^55^^,^^56^ to be associated with lower treatment success.

The fact that sex and health insurance type were not significant predictors of treatment outcome in the incarcerated population, however, is likely due to the low variation in these factors in this population (most incarcerated people being male and having public health insurance).

Regarding adherence as a predictor of treatment success, although our observed protective effect of adherence is expected, the strong effect observed here may be an underestimation of the true effect, given that expected doses were based on initial regimen assigned, and therefore inadequately reflect adherence in patients whose treatment was extended (who are also more likely to have had poorer outcomes). In addition, the fact that the proportion of those with good adherence (≥80% of expected doses taken) was lower in those with a missing outcome compared to those with an outcome available (in the non-incarcerated population) could potentially bias results, given that those with low adherence are expected to also have a lower likelihood of treatment success. However, as sensitivity analyses showed that exclusion of those with missing outcome vs. their recategorization as having had an unsuccessful outcome did not significantly alter estimates, this is not expected to have biased the findings.

Comparing our findings to other South American countries, aside from Peru, Brazil is the only other South American country identified by the WHO as among the top 16 contributors to the global shortfall in TB case notifications in 2020 (vs. 2019).^3^ An ITS study in Brazil, also comparing pre- and during-COVID-19 trends in TB case notifications similarly found an increasing trend in TB notifications pre-COVID-19, and a decreasing trend between 2010 and 2021 (i.e., including the pandemic period). The monthly percent decrease in case notifications in this period (2010–2021) was found to be 8.10% (95% CI: 0.54%–15.08%).^57^ Another Brazilian study, comparing TB case notifications in January to July 2019 vs. January to July 2020 in one state in Brazil (Bahia) found a 26.4% decrease in notifications in the latter period.^58^ Although these studies and our own echo global findings regarding the impact of COVID-19 on TB,^3^^,^^59^ comparison across settings and studies is difficult due to the heterogeneity of methods used and of COVID-19 period definitions.

Our study has several limitations. One limitation is that although we have investigated the impact of COVID-19 on TB case notifications and outcomes in the incarcerated and non-incarcerated populations of Peru, it is difficult to draw conclusions about the mechanisms behind this impact. For example, the fact that TB case notifications dropped during COVID-19 is expected to be a result of disruptions to TB diagnosis and reporting services, rather than representing an actual decrease in TB incidence, however, the specific mechanisms contributing to fewer TB diagnoses being captured in this period (e.g., changes in care-seeking behaviour vs. health systems disruptions, etc.) cannot be elucidated from this study.

Unfortunately, lacking data on symptom onset, we could not explore diagnostic delays (i.e., time from symptom onset to diagnosis). A suitable indicator of disease severity was also unavailable in SIGTB and could therefore not be used as a proxy for diagnostic delay. We therefore could not explore whether earlier diagnosis acted as a mediator of the effect of COVID-19 period on TB treatment success. Earlier diagnosis in prisons compared to in the non-incarcerated population however is possible, given that detainees receive a physical health evaluation at entry, and that detainee health promoters and peer-supporters assist in identifying TB symptoms early among cellmates. In addition, given that incarcerated populations are generally younger than the overall population, it is possible that competing risks, such as COVID-19 mortality, differentially affected the incarcerated and non-incarcerated populations and subsequently their TB outcomes, but it is difficult to separate this effect from other influences like increased COVID-19 transmission in prisons.

Other limitations of our study include challenges inherent in the use of secondary data collected for routine programmatic purposes. This includes the fact that cases are often under-notified in national TB program databases,^60^^,^^61^ including SIGTB,^62^ with notified TB cases in Peru representing 81.4% of the estimated national burden prior to COVID-19 (in 2019)^63^ and 60.9% of the estimated burden in 2020.^63^ This suggests that estimates of the effect of receiving TB treatment during vs. prior to COVID-19 on treatment success could be biased towards the null, if patients with poorer treatment outcomes were less likely to be notified during the pandemic.

Another limitation is the lack of a dynamic indicator of incarceration status in national TB program data. As incarceration is only captured in the SIGTB database for those who received a TB diagnosis while incarcerated, the present analysis could not consider prior incarceration, incarceration after case notification, or length of incarceration. Although the majority of incarcerated individuals in Peru serve sentences longer than 5 years,^64^ individual-level data on length of stay would have allowed a more precise—and dynamic–definition of incarceration status in the analysis. In addition, other data limitations necessitated reliance on imperfect indicators. For example, the lack of data on individuals’ socioeconomic status meant that health insurance type was used as an imperfect proxy. Similarly, our estimates of the effect of adherence on treatment outcomes are limited due to having information only on the number of doses at exit, but not the proportion of doses missed throughout an individual’s treatment. As a result, the adherence proportion was estimated based on individuals’ total doses expected for their treatment regimen and their time on treatment before exit, possibly leading to imperfect classification of true adherence.

Strengths of our study include its size and representativeness of all case notifications in the country. In addition, we sought to underline the urgent yet under-studied public health concern of TB in prisons, highlighting the impact of this disease in a neglected population. Prisons must not be overlooked in the overall TB response, firstly because the right to health extends to individuals experiencing incarceration, as it does to everyone. Incarcerated populations should therefore not be subjected to conditions that put their health at much higher risk than the general population. Secondly, our analysis suggests a lower extent of COVID-related TB care disruptions in prisons, suggesting that TB services among the incarcerated population were less sensitive to COVID-19-related disruptions than TB services in the non-incarcerated population. This resilience of TB services in the carceral setting to external disruptions, in combination with the high burden of TB in this population, highlights prisons as unique settings in which TB prevention and treatment is feasible yet urgent, making clear that prisons should be key priorities for TB elimination efforts in Peru and elsewhere.

## Contributors

Study conceptualisation: CUG, LM, MP, LF. Data curation and analysis/visualisation: LF, SH. Study supervision: CUG, MP, LM, SH. Writing of original draft: LF. Review and editing: CUG, MP, LM, SH, AB, GCC, JRV, RCT, MCA. Access and verification of all data reported in the study: LF, CUG. Responsible for the decision to submit for publication: LF, CUG. All authors agreed with the decision to submit for publication.

## Data sharing statement

The data used in these analyses are from the Peruvian National TB Program database and cannot be shared. Access to these data must be requested from and approved by the Peruvian NTP.

## Editor note

The Lancet Group takes a neutral position with respect to territorial claims in published maps and institutional affiliations.

## Declaration of interests

MP serves as an advisor to several non-profit organizations including Bill & Melinda Gates Foundation, WHO, Stop TB Partnership and FIND. He has no financial or industry conflicts. CUG has received research support from the International Development Research Centre (Canada) and the Canadian Institutes of Health Research, the National Institutes of Health, FIND, and Abbott for projects unrelated to this work. CUG has also received honoraria from Molbio and Abbott for presentations unrelated to this work. SH was supported by a Canadian Institute of Health Research Postdoctoral Fellowship. LF was supported by a Canadian Institutes of Health Research Doctoral Award, and subsequently by a Banting Postdoctoral Fellowship. The other authors declared no conflicts of interest.

## Appendix ASupplementary data

Supplementary Materials

Spanish_abstract_disclaimer
