# Supplementary material for: Tuberculosis case notifications and outcomes in Peruvian prisons prior to and during the COVID-19 pandemic: a national-level interrupted time series analysis
Source: Lancet Reg Health Am. 2024 Mar 27;33:100723. doi: 10.1016/j.lana.2024.100723 (PMC11117008; doi:10.1016/j.lana.2024.100723)
Supplement: Supplementary Materials [file mmc1.pdf]

Supplementary material for: Tuberculosis case notifications and outcomes in Peruvian prisons prior to and during the COVID-19 pandemic: A national-level interrupted time series analysis

Lena Faust,[1,2,3] Guillermo Caceres-Cardenas,[4] Leonardo Martinez,[5] Sophie Huddart,[6,7] Julia Rios Vidal,[8] Ronald Corilloclla-Torres,[9] Mayra Cordova Ayllon,[10] Andrea Benedetti,[1] Madhukar Pai,[1,2] César Ugarte-Gil[4,11,12]

Affiliations:

1. Department of Epidemiology, Biostatistics and Occupational Health, McGill University, Montréal, Canada
2. McGill International TB Centre, McGill University, Montréal, Canada
3. London School of Hygiene and Tropical Medicine, London, United Kingdom
4. Instituto de Medicina Tropical Alexander von Humboldt, Universidad Peruana Cayetano Heredia, Lima, Perú
5. School of Public Health, Boston University, Boston, USA
6. Division of Pulmonary and Critical Care Medicine, University of California San Francisco, San Francisco, USA
7. UCSF Center for Tuberculosis, University of California San Francisco, San Francisco, USA
8. Dirección De Control y Prevención de la Tuberculosis (DPCT), Ministerio de Salud, Lima, Perú
9. Subdireción de Salud Penitenciaria, Instituto Nacional Penitenciario, Lima, Perú
10. Estrategia de Tuberculosis, Instituto Nacional Penitenciario, Lima, Perú
11. School of Medicine, Universidad Peruana Cayetano Heredia, Lima, Perú
12. University of Texas Medical Branch, Galveston, USA

Supplementary Material

Supplement Section 1 (DS-TB notifications by department, pre- and during-COVID-19):

Incidence Rate Ratio (IRR) calculations and data sources:

$$\text{IRR} = \frac{\text{DS−TB case notifications per 100,000 in incarcerated population of department}}{\text{DS−TB case notifications per 100,000 in total population of department}}$$

Denominators for incidence in incarcerated population:

- Pre-COVID-19: average incarcerated population of department, Mar 2019 to Feb 2020 (from publicly available INPE data).[1]
- During COVID-19: average incarcerated population of department, Mar 2020 to Feb 2021 (from publicly available INPE data).[1]

Denominators for incidence in total population:

- Pre-COVID-19: census projection for overall 2019 department population[2]
- During COVID-19: census projection for overall 2020 department population[2]

Table S1. Notification rates and *IRRs for DS-TB in the prison vs. total population in Peru, by department*

Department	Pre-COVID-19 (a)			During COVID-19 (a)		
	Notification rate (per 100,000 people) in overall population	Notification rate (per 100,000 people) in incarcerated population (b)	IRR (c)	Notification rate (per 100,000 people) in overall population	Notification rate (per 100,000 people) in incarcerated population (b)	IRR (c)
**Amazonas**	21.00	0.00	0.00	13.36	0.00	0.00
**Ancash**	55.92	2234.52	39.96	41.16	2305.94	56.02
**Apurimac**	18.39	0.00	0.00	9.75	0.00	0.00
**Arequipa**	53.53	1325.43	24.76	34.39	1393.19	40.51
**Ayacucho**	32.36	0.00	0.00	17.21	0.00	0.00
**Cajamarca**	14.71	296.74	20.17	10.18	107.12	10.52
**Callao**	133.33	0.00	0.00	86.03	0.00	0.00
**Cusco**	33.05	235.73	7.13	21.44	221.55	10.33
**Huancavelica**	27.47	0.00	0.00	9.03	0.00	0.00
**Huanuco**	58.83	1127.93	19.17	39.46	441.36	11.19
**Ica**	124.93	4218.36	33.76	89.21	3772.82	42.29
**Junin**	50.22	99.98	1.99	34.15	189.50	5.55
**La Libertad**	70.10	2181.38	31.12	44.43	1966.69	44.27
**Lambayeque**	83.04	8331.50	100.33	40.36	2360.02	58.48
**Lima**	145.90	5170.59	35.44	98.41	4143.41	42.10
**Loreto**	126.38	0.00	0.00	98.78	0.00	0.00
**Madre de Dios**	133.59	921.19	6.90	109.31	209.42	1.92
**Moquegua**	83.78	0.00	0.00	66.41	0.00	0.00
**Pasco**	20.58	0.00	0.00	15.81	0.00	0.00
**Piura**	28.95	1165.83	40.26	18.70	1145.62	61.26
**Puno**	32.77	84.46	2.58	21.41	130.83	6.11
**San Martin**	44.67	1255.23	28.10	30.68	1287.13	41.96
**Tacna**	116.46	1867.70	16.04	88.15	1995.01	22.63
**Tumbes**	53.91	2602.85	48.28	45.72	3254.97	71.19
**Ucayali**	182.07	1915.56	10.52	118.82	2300.67	19.36
**PERU**	**89.42**	**2894.46**	**32.37**	**60.14**	**2244.63**	**37.32**

a) Pre-COVID-19 = DS-TB diagnosis between 15 March 2019 and 15 March 2020. During COVID-19 = DS-TB diagnosis between 16 March 2020 (start of COVID-19 restrictions in Peru) and 16 March 2021.

b) *0 rates indicate that no TB case was notified in that region and group during the relevant period.*
c) *IRR = incidence rate ratio = notification rate in prisons / notification rate in department*

Supplement Section 2 (Interrupted time series of weekly DS-TB case notifications):

Equation S1: Negative binomial regression for weekly DS-TB case notifications in the *total population*:

$$log(TB\_cases\_total) = \beta_0 + \beta_1 X_{phase2} + \beta_2 X_{phase3} + \beta_3 X_{time} + \beta_4 X_{phase1:sin\_phase1} + \beta_5 X_{phase2:time} + \beta_6 X_{phase3:time} + \beta_7 X_{phase3:sin\_phase3} + log(population\_total)$$

Where:

- *TB_cases_total = weekly DS-TB case notifications in the total population of Peru*
- *Phase 1 = pre-COVID-19: weeks 1-115 (COVID-19 national state of emergency declared in week 116). Coded 1 in weeks 1-115, 0 otherwise.*
- *Phase 2 = recovery period (linear increase following initial drop in cases), weeks 116-151. Coded 1 in weeks 116-151, 0 otherwise.*
- *Phase 3 = post-recovery period (resumption of harmonic trend similar to pre-COVID-19), weeks 152 onwards. Coded 1 in weeks 152-209, 0 otherwise.*
- *Time = Weeks (1 to 209)*
- *Sin_phase1 = harmonic trend for phase 1, sin(3πTime/115). (See **Figure S1**).*
- *Sin_phase3 = harmonic trend for phase 3, sin(2πTime/58). (See **Figure S1**).*
- *Population_total = the total population size, based on annual (2018, 2019, 2020 and 2021) population projections from the Peruvian census[3]*

Equation S2: Negative binomial regression for weekly DS-TB case notifications in the *non-incarcerated population*:

$$log(TB\_cases\_noninc) = \beta_0 + \beta_1 X_{phase2} + \beta_2 X_{phase3} + \beta_3 X_{time} + \beta_4 X_{phase1:sin\_phase1} + \beta_5 X_{phase2:time} + \beta_6 X_{phase3:time} + \beta_7 X_{phase3:sin\_phase3} + log(population\_noninc)$$

Where:

- *TB_cases_noninc* = weekly DS-TB case notifications in the non-incarcerated population of Peru
- *Phase 1 = pre-COVID-19: weeks 1-115 (COVID-19 national state of emergency declared in week 116). Coded 1 in weeks 1-115, 0 otherwise.*
- *Phase 2 = recovery period (linear increase following initial drop in cases), weeks 116-151. Coded 1 in weeks 116-151, 0 otherwise.*
- *Phase 3 = post-recovery period (resumption of harmonic trend similar to pre-COVID-19), weeks 152 onwards. Coded 1 in weeks 152-209, 0 otherwise.*
- *Time = Weeks (1 to 209)*
- *Sin_phase1 = harmonic trend for phase 1, sin(3πTime/115). (See **Figure S2**).*
- *Sin_phase3 = harmonic trend for phase 3, sin(2πTime/58). (See **Figure S2**).*

- *Population_noninc* = the non-incarcerated population size. The non-incarcerated population size is based on annual (2018, 2019, 2020 and 2021) population projections from the Peruvian census,[3] minus the average annual incarcerated population, from Peru's penitentiary institute database[1]

Equation S3: Negative binomial regression for weekly DS-TB case notifications in the *incarcerated population*:

$$\log(TB\_cases\_incarc) = \beta_0 + \beta_1 X_{COVID-19} + \beta_2 X_{Time} + \beta_3 X_{COVID:Time} + \beta_4 X_{t.sin} + \log(population\_incarc)$$

Where:

- *TB_cases_incarc = weekly DS-TB case notifications in the incarcerated population of Peru*
- *COVID-19 = indicator variable for COVID-19 period, coded 0 pre-COVID (weeks 1 to 115) and coded 1 during COVID (weeks 116 to 209).*
- *Time = weeks (1 to 209)*
- *t.sin = harmonic trend, sin(2πTime/209). (See **Figure S3**).*
- *Population_incarc = the incarcerated population size, based on monthly counts of the incarcerated population from Peru's penitentiary institute database[1]*

Figure S1. *Total number of people experiencing incarceration in Peru, by month, 2018-2021*

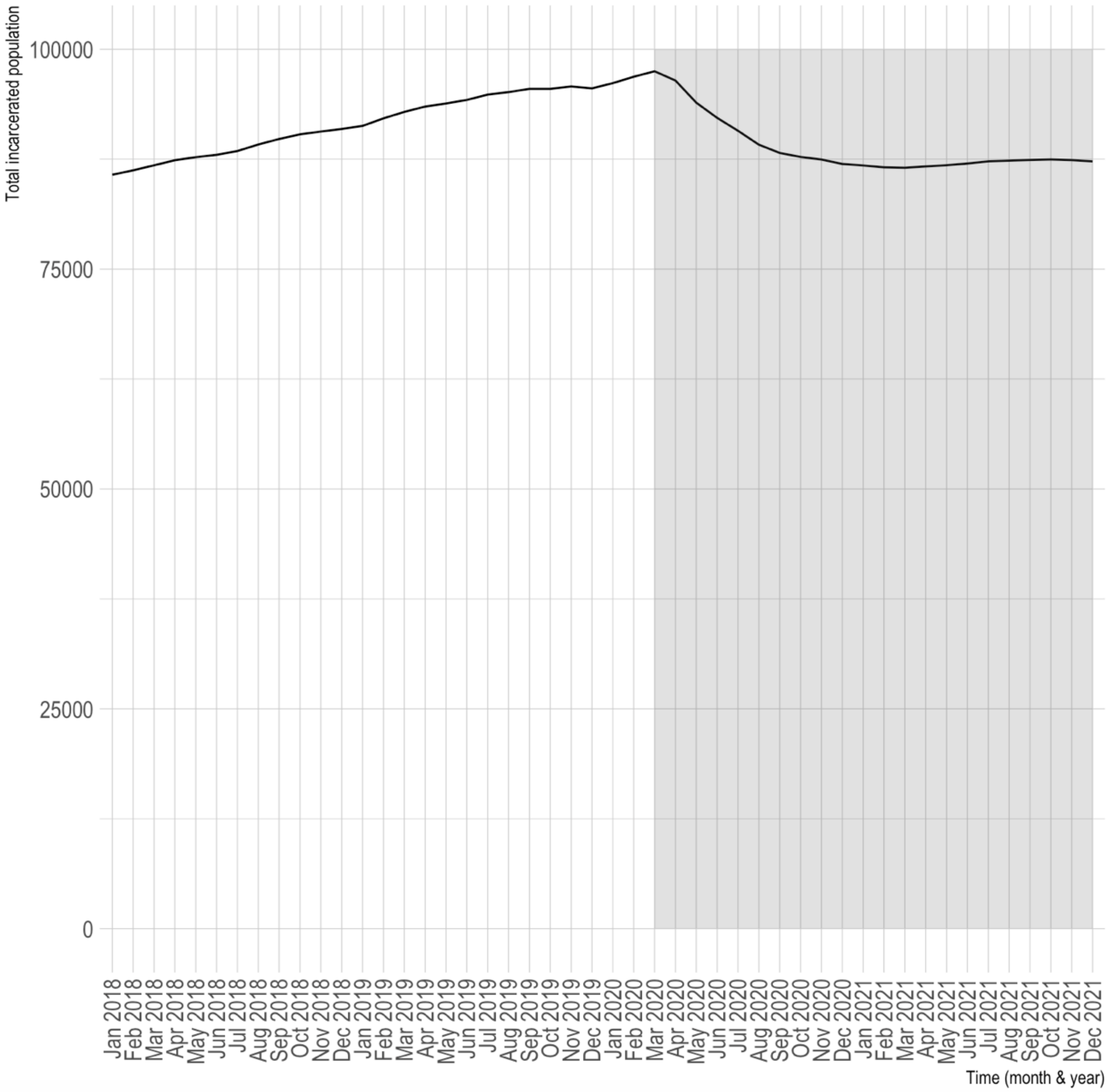

Population estimates from Peruvian National Penitentiary Institute (INPE)[1]
Shaded area: COVID-19 pandemic period

Observed values vs. fitted trend and detrended residuals for TB case notifications over time
Figures S2-S4 show the harmonic terms used to model time in the non-linear phases, and the corresponding detrended residuals. Harmonic terms were defined as appropriate to the trend, e.g. $\sin(\frac{w \times \pi \times time}{total\ time})$ where w is the number of waves in the phase, $time$ is the week number (e.g. from 1 to 209) and $total\ time$ is the total number of weeks in the phase.

In addition to the detrended residuals, Durbin-Watson (DW) test results of 1.91 (p=0.267), 1.89 (p=0.216), and 2.03 (p=0.574) in the total population, non-incarcerated population, and incarcerated population models, respectively, indicate no significant autocorrelation (DW values close to 2 and p>0.05 suggest absence of significant autocorrelation).

Figure S2: Observed values vs. fitted trend (left) and detrended residuals (right) for the total population model (non-incarcerated and incarcerated)

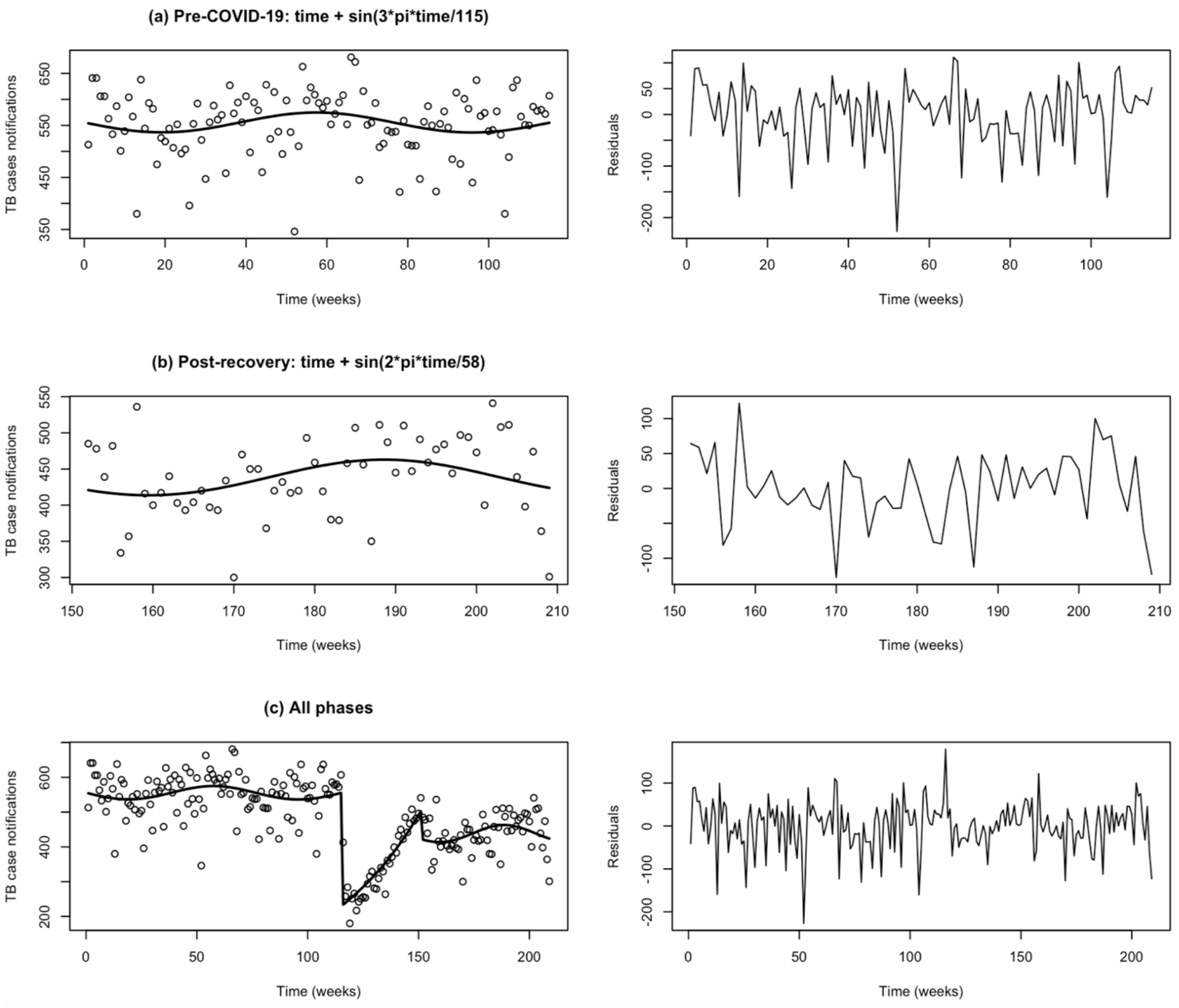

Phase 1: Pre-COVID-19 (Week 1 to 115)
Phase 2: During COVID-19, recovery phase (Week 116 to 151)
Phase 3: During COVID-19, post-recovery phase (Week 151 to 209)

Figure S3: Observed values vs. fitted trend (left) and detrended residuals (right) for the non-incarcerated population model

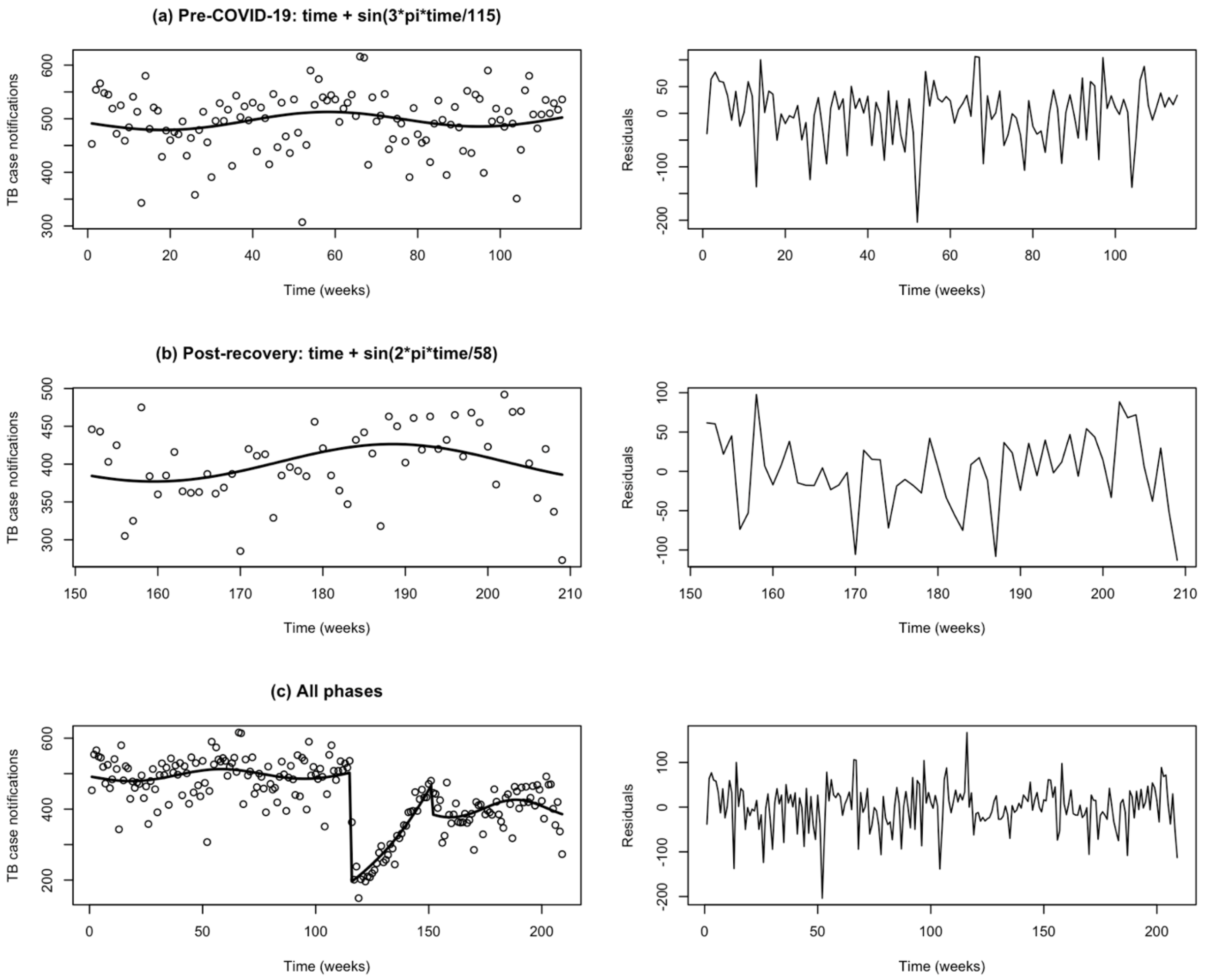

Phase 1: Pre-COVID-19 (Week 1 to 115)
Phase 2: During COVID-19, recovery phase (Week 116 to 151)
Phase 3: During COVID-19, post-recovery phase (Week 151 to 209)

Figure S4: Observed values vs. fitted trend (left) and detrended residuals (right) for the incarcerated population model

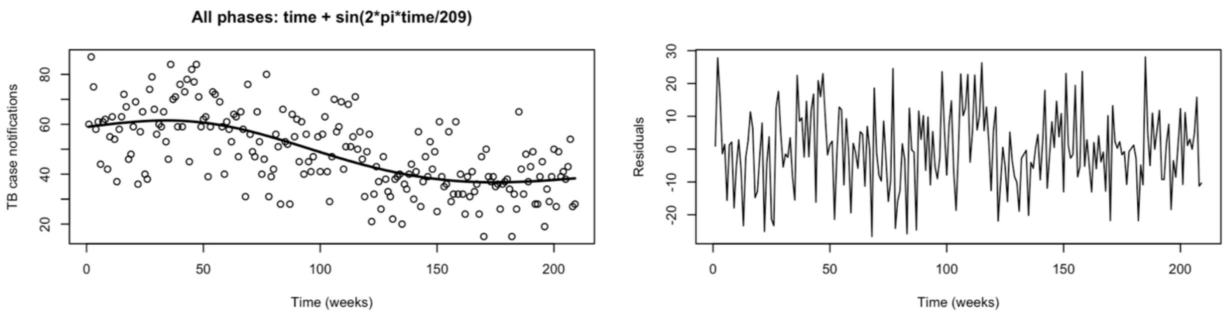

Figure S5. *DS-TB case notification rates (per 100,000 population) in Peru pre- and during COVID-19, 2018-2021.*

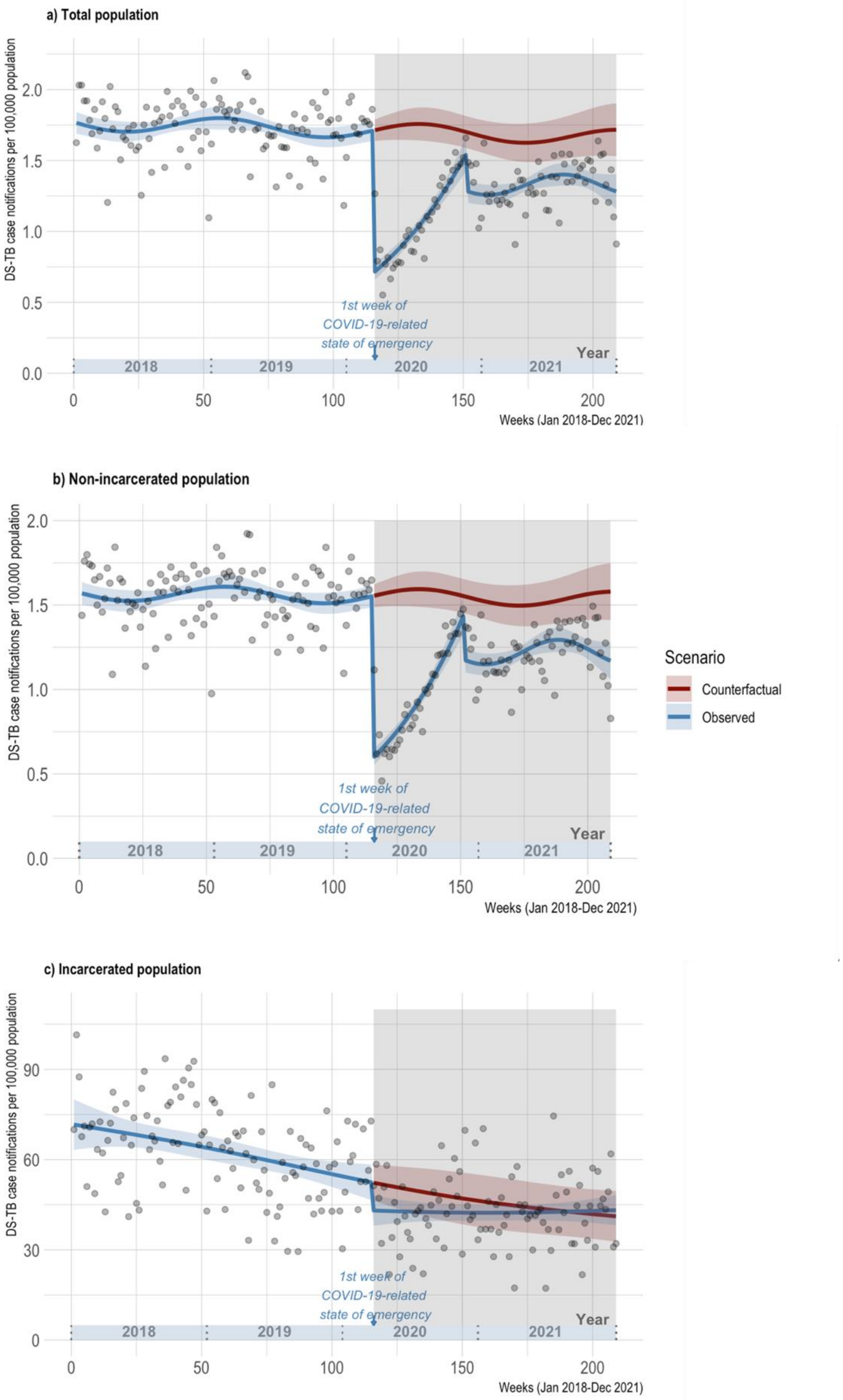

Table S2. *Linear component of trends in DS-TB case notifications in Peru pre- and during the COVID-19 pandemic*

	**Phase 1**			**Phase 2**			**Phase 3**		
**Total population (a)**									
	Incidence rate ratio (b)	95%CI		Incidence rate ratio (b)	95%CI		Incidence rate ratio (b)	95%CI	
Per week	1.00	1.00	1.00	1.02	1.02	1.03	1.00	1.00	1.00
Per month	1.00	1.00	1.00	1.09	1.07	1.11	1.00	0.99	1.01
**Non-incarcerated population (a)**									
	Incidence rate ratio (b)	95%CI		Incidence rate ratio (b)	95%CI		Incidence rate ratio (b)	95%CI	
Per week	1.00	1.00	1.00	1.03	1.02	1.03	1.00	1.00	1.00
Per month	1.00	1.00	1.00	1.10	1.09	1.12	1.00	0.99	1.01
**Incarcerated population (c)**									
	**Pre-COVID-19**			**During COVID-19**					
	Incidence rate ratio (d)	95%CI		Incidence rate ratio (d)			95%CI		
Per week	1.00	1.00	1.00	1.00			1.00	1.00	
Per month	0.99	0.98	0.99	1.00			0.99	1.01	

(a) Phases in the non-incarcerated and total population models:
Phase 1 = pre-COVID-19: weeks 1-115 (COVID-19 national state of emergency declared in week 116). Coded 1 in weeks 1-115, 0 otherwise. Phase 2 = recovery period (linear increase following initial drop in cases), weeks 116-151. Coded 1 in weeks 116-151, 0 otherwise. Phase 3 = post-recovery period (resumption of harmonic trend similar to pre-COVID-19), weeks 152 onwards. Coded 1 in weeks 152-209, 0 otherwise.
(b) Slope for linear component of trend in each phase, i.e., increase in cases per week in each phase (where 'time' = weeks [1-209]): Phase 1: $e^{\beta_{time}}$. *Phase 2:* $e^{(\beta_{time} + \beta_{phase\ 2:time})}$. *Phase 3:* $e^{(\beta_{time} + \beta_{phase\ 3:time})}$.
*See **Supplement Equations S1 and S2** for full models, and **Supplement Figures S1 and S2** for harmonic terms.*
(c) Phases in the incarcerated population model: During COVID-19: Indicator for COVID-19, equal to 0 prior to COVID-19 and equal to 1 in first week of COVID-19 restrictions and thereafter (as of week 116, declaration of COVID-19-related national state of emergency).
(d) Slope for linear component of trend pre-COVID-19 and during COVID-19, i.e., increase in cases per week in each phase (where 'time' = weeks [1-209]): Pre-COVID-19: $e^{\beta_{time}}$. *During COVID-19:* $e^{(\beta_{time} + \beta_{covid:time})}$.
*See **Supplement Equation S3** for full model, and **Supplement Figure S3** for harmonic term.*

In the total and non-incarcerated population, cases increased by 2% (95%CI: 2-3%) each week in Phase 2 (IRR: 1.02, 95%CI: 1.02-1.03), the recovery period in which cases increased gradually after the large initial drop in the first week of COVID-19. In Phase 3 (post-recovery), the trend in cases returned to having no significant change in cases per week (IRR: 1.00, 95%CI: 1.00-1.00), similar to the pre-COVID-19 trend. In the incarcerated population, there was a 1% decrease in case notifications per week in the pre-COVID-19 period (95%CI: 1-2%), and no change in cases per week in the during-COVID-19 period.

Table S3. Percent differences in weekly observed vs. counterfactual DS-TB case notification rates (per 100,000 people) in the incarcerated and non-incarcerated populations of Peru at 3-month time points during the COVID-19 pandemic

Year	Week (2nd week of:)	Week number (a)	Scenario	Total			Non-incarcerated			Incarcerated		
				rate	95%CI		rate	95%CI		rate	95%CI	
**2020**	**Mar**	116	Observed	0.7	0.7	0.8	0.6	0.6	0.7	43.1	38.0	48.1
			Counterfactual	1.7	1.6	1.8	1.6	1.5	1.6	52.3	46.3	58.3
			% Difference (b)	-58.1	-59.7	-56.7	-61.2	-62.7	-59.9	-17.7	-17.9	-17.5
	**Jun**	129	Observed	1.0	0.9	1.0	0.8	0.8	0.9	42.7	39.1	46.3
			Counterfactual	1.8	1.6	1.9	1.6	1.5	1.7	50.1	42.8	57.5
			% Difference (b)	-45.7	-44.7	-46.6	-47.7	-46.8	-48.6	-14.8	-8.5	-19.5
	**Sep**	142	Observed	1.3	1.2	1.3	1.1	1.1	1.2	42.5	39.6	45.3
			Counterfactual	1.7	1.6	1.9	1.6	1.5	1.7	48.2	39.9	56.5
			% Difference (b)	-27.3	-26.1	-28.5	-27.6	-26.3	-28.7	-11.9	-0.6	-19.8
	**Dec**	155	Observed	1.3	1.2	1.3	1.2	1.1	1.2	42.3	39.7	45.0
			Counterfactual	1.7	1.6	1.8	1.5	1.4	1.6	46.4	37.8	55.1
			% Difference (b)	-24.7	-24.0	-25.3	-24.7	-24.0	-25.3	-8.8	5.2	-18.5
**2021**	**Mar**	168	Observed	1.3	1.2	1.3	1.2	1.1	1.2	42.4	39.8	45.0
			Counterfactual	1.6	1.5	1.8	1.5	1.4	1.6	44.9	36.3	53.6
			% Difference (b)	-21.2	-18.1	-23.9	-21.6	-18.6	-24.3	-5.7	9.7	-16.1
	**Jun**	181	Observed	1.4	1.3	1.4	1.3	1.2	1.3	42.5	39.8	45.3
			Counterfactual	1.6	1.5	1.8	1.5	1.4	1.6	43.6	35.2	52.0
			% Difference (b)	-15.6	-12.2	-18.6	-15.6	-12.2	-18.4	-2.4	13.0	-12.9
	**Sep**	194	Observed	1.4	1.3	1.5	1.3	1.2	1.3	42.8	39.3	46.3
			Counterfactual	1.7	1.5	1.8	1.5	1.4	1.7	42.4	34.2	50.6
			% Difference (b)	-17.4	-13.3	-20.8	-17.2	-13.2	-20.5	0.9	15.0	-8.6
	**Dec**	207	Observed	1.3	1.2	1.4	1.2	1.1	1.3	43.1	38.4	47.9
			Counterfactual	1.7	1.5	1.9	1.6	1.4	1.7	41.3	32.9	49.7
			% Difference (b)	-24.5	-22.6	-26.1	-25.0	-23.1	-26.5	4.4	16.5	-3.6

(a) week 1 = week of 1 Jan 2018. Week 116 = first week of COVID-19 (mid-March 2020)

*(b) % difference = (observed rate - counterfactual rate) / counterfactual rate * 100*

i.e., Negative sign indicates a drop in notifications during COVID-19 compared to expected value (counterfactual)

% Difference in notification rate:

-60%

0%

20%

Supplement Section 3 (DS-TB treatment success, pre- and during-COVID-19):

Table S4. *Summary of characteristics of DS-TB patients in Peru with available vs. missing treatment outcome data:*

	Total population		Non-incarcerated population		Incarcerated population	
	**Outcome available**	**Outcome missing**	**Outcome available**	**Outcome missing**	**Outcome available**	**Outcome missing**
**Total**	N = 80,535	N = 2,364	N = 71,981	N = 2,311	N = 8,554	N = 53
**Age (years)**	40.3 (18.1)	42.8 (18.2)	41.2 (18.5)	42.9 (18.3)	32.2 (10.4)	35.9 (13.7)
**Sex, male**	52,732 (65.5%)	1,462 (61.8%)	44,224 (61.4%)	1,409 (61.0%)	8,508 (99.5%)	53 (100.0%)
**Extrapulmonary TB**	15,609 (19.4%)	712 (30.1%)	14,789 (20.5%)	701 (30.3%)	820 (9.6%)	11 (20.8%)
**Type of case**						
New	70,238 (87.2%)	2,136 (90.4%)	64,225 (89.2%)	2,090 (90.4%)	6,013 (70.3%)	46 (86.8%)
Previous treatment stopped or failed	2,405 (3.0%)	54 (2.3%)	2,287 (3.2%)	54 (2.3%)	118 (1.4%)	0 (0.0%)
Relapse	7,892 (9.8%)	174 (7.4%)	5,469 (7.6%)	167 (7.2%)	2,423 (28.3%)	7 (13.2%)
**HIV positive**	4,240 (5.7%)	132 (6.6%)	3,971 (6.0%)	131 (6.8%)	269 (3.2%)	1 (2.0%)
**Diabetes**	7,279 (9.8%)	234 (11.1%)	7,067 (10.4%)	230 (11.1%)	212 (3.4%)	4 (9.1%)
**Smoking**	7,156 (8.9%)	94 (4.0%)	3,110 (4.3%)	81 (3.5%)	4,046 (47.3%)	13 (24.5%)
**Type of health insurance**						
Private	293 (0.4%)	8 (0.3%)	293 (0.4%)	8 (0.3%)	0 (0.0%)	0 (0.0%)
Employer's (a)	16,545 (20.6%)	1,071 (45.5%)	16,530 (23.0%)	1,071 (46.5%)	15 (0.2%)	0 (0.0%)
Public (b)	54,237 (67.5%)	1,179 (50.1%)	45,769 (63.8%)	1,126 (48.9%)	8,468 (99.1%)	53 (100.0%)
None	9,259 (11.5%)	97 (4.1%)	9,196 (12.8%)	97 (4.2%)	63 (0.7%)	0 (0.0%)
**COVID-19 period**						
Pre-COVID-19 TB care	47,133 (58.5%)	802 (33.9%)	41,986 (58.3%)	791 (34.2%)	5,147 (60.2%)	11 (20.8%)
TB care fully during COVID-19	18,355 (22.8%)	1,230 (52.0%)	16,369 (22.7%)	1,189 (51.4%)	1,986 (23.2%)	41 (77.4%)
TB care partially during COVID-19	15,044 (18.7%)	332 (14.0%)	13,623 (18.9%)	331 (14.3%)	1,421 (16.6%)	1 (1.9%)
**Good adherence (>=80% of expected doses taken)**	72,342 (92.3%)	120 (78.9%)	64,065 (91.6%)	116 (78.4%)	8,277 (98.1%)	4 (100.0%)

(a) Seguro Social de Salud (EsSalud) or police/armed forces *(b) Seguro Integral de Salud (SIS)*
Missing data on covariates (in total population): Extrapulmonary/pulmonary TB: n=12, HIV: n=6,269, diabetes: n=6,866, smoking: n=138, health insurance type: n=210, COVID-19 period: n=3, adherence: n=4,343.

Equation S4
Logistic regression for DS-TB treatment outcomes:
(Separate models for the total, incarcerated, and non-incarcerated populations)

$$logit\big(p(Y=1|\boldsymbol{X})\big) = \beta_0 + \beta_1 X_{COVID-19\_full} + \beta_2 X_{COVID-19\_partial} + spline(X_{age}) + \beta_3 X_{sex} + \beta_4 X_{HIV} + \beta_5 X_{diabetes} + \beta_6 X_{EPTB} + \beta_7 X_{type\_relapse} + \beta_8 X_{type\_previous} + \beta_9 X_{smoking} + \beta_{10} X_{insurance\_employer} + \beta_{11} X_{insurance\_public} + \beta_{12} X_{insurance\_none} + \beta_{13} X_{adherence}$$

Where:
- *Y=1:* DS-TB treatment success (cure or treatment complete)
- *COVID-19_full:* TB care fully during the COVID-19 pandemic (vs. pre-COVID-19)
- *COVID-19_partial:* TB care partially during the COVID-19 pandemic (vs. pre-COVID-19)
- *EPTB:* extrapulmonary TB (vs. pulmonary TB)
- *Type_relapse:* Type of case: relapse (vs. new case)
- *Type_previous:* Type of case: previous TB treatment stopped or failed (vs. new case)
- *Insurance_employer:* Employer's health insurance (vs. private insurance)
- *Insurance_public:* Public health insurance (vs. private insurance)
- *Insurance_none:* No health insurance (vs. private insurance)
- *Adherence:* >= 80% of expected doses taken (vs. <80%)

Imputation diagnostics:

Multiple imputation by chained equations (MICE) was used to impute missing values for covariates. Counts across strata of the imputed categorical variables (HIV status, extrapulmonary vs pulmonary TB, diabetes, adherence, covid period, type of health insurance, and smoking) were similar between the original and imputed datasets (chi-squared p values ranging from 0.095 to >0.999 for the aforementioned variables), suggesting imputations were successful (the distribution of the imputed data does not differ significantly from the original). For the continuous covariate (age), the Kolmogorov-Smirnov (K-S) test indicated that the imputed datasets originated from the same distribution as the original (all K-S test p values >0.999 for comparisons of original vs. imputed age data for the first five imputed datasets of all three models (all, incarcerated, and non-incarcerated).

Effect of COVID-19 period on TB treatment success: Risk ratios

Using predicted probabilities derived from the logistic models (**Eq S4**) in the total, incarcerated and non-incarcerated populations, risk ratios for the effect of COVID-19 period on TB treatment outcomes (with other covariates held at fixed values) are shown in **Table S5**. The constant values for covariates were: age = 40 years, sex = male, HIV = negative, diabetes = no, smoking = no, adherence category >= 80% of doses taken, insurance type = public, case type = new, site of TB = pulmonary). Therefore, the probabilities below do not represent treatment success probabilities for the overall population, but for a new case of pulmonary TB in a 40-year-old,

HIV-negative, non-diabetic, non-smoking, >=80% adherent male with public health insurance. In all populations, risk of **unsuccessful** treatment was higher in those receiving care during COVID-19 (e.g., RR for treatment success in the total population: 0.97 (95%CI: 0.96-0.97).

Table S5. Risk ratios for the effect of COVID-19 on TB treatment success in the total, incarcerated and non-incarcerated population

	**Total population**	**Non-incarcerated population**	**Incarcerated population**
Probability of treatment success, **Pre-COVID TB care**[a] (95%CI)	0.85 (0.84-0.85)	0.84 (0.83-0.85)	0.88 (0.86-0.90)
Probability of treatment success, fully **during-COVID TB care**[a] (95%CI)	0.82 (0.81-0.83)	0.81 (0.80-0.82)	0.85 (0.82-0.87)
**Risk Ratio (pre- vs. during-COVID)**[a] (95%CI)	0.97 (0.96-0.97)	0.96 (0.96-0.97)	0.96 (0.96-0.97)

(a) Treatment success probabilities are for a new case of pulmonary TB in a 40-year-old, HIV-negative, non-diabetic, non-smoking, >=80% adherent male with public health insurance.

Interaction between COVID-19 period and incarceration status:

Table S6. *Effect of COVID-19, incarceration, and other covariates*[a,b] *on DS-TB treatment success, including a COVID-19*incarceration interaction term*

	**OR**	**95%CI**	
**COVID-19 period: (Ref=TB care pre-COVID-19)**			
**TB care fully during COVID-19**	0.81	0.78	0.85
**TB care partially during COVID-19**	0.93	0.89	0.98
**Incarcerated (Ref = non-incarcerated)**	1.52	1.39	1.67
**Sex, male (Ref = female)**	0.78	0.75	0.81
**HIV positive**	0.44	0.41	0.48
**Diabetes**	0.99	0.93	1.05
**Extrapulmonary TB**	1.41	1.34	1.48
**Type of case: (Ref=new case)**			
**Previous treatment stopped or failed**	0.40	0.36	0.44
**Relapse**	0.74	0.69	0.78
**Smoking**	0.72	0.68	0.78
**Insurance type: (Ref=private insurance)**			
**Employer's insurance (c)**	0.75	0.53	1.07
**Public insurance (d)**	0.64	0.46	0.91
**None**	0.69	0.49	0.98

**Good adherence (>=80% of expected doses taken)**	7.72	7.28	8.18
**Interaction, COVID-19 period & incarceration status**			
**TB care fully during COVID-19 : incarcerated**	1.07	0.92	1.25
**TB care partially during COVID-19 : incarcerated**	1.03	0.87	1.22

(a) Effect of age was non-linear and modelled using splines
(b) Missing covariate data imputed via multiple imputation by chained equations (MICE)
(c) Seguro Social de Salud (EsSalud) or police/armed forces
(d) Seguro Integral de Salud (SIS)

Sensitivity Analyses:

Figure S6. *Comparison of adjusted odds ratios for treatment success using complete case analysis vs. multiple imputation by chained equations for missing covariate data*

Model 1: All

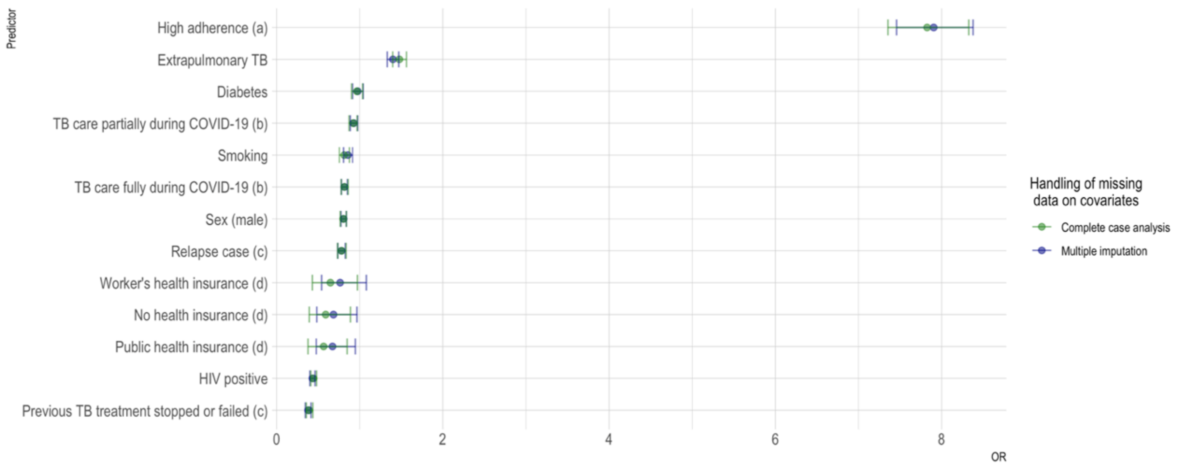

Model 2: non-incarcerated

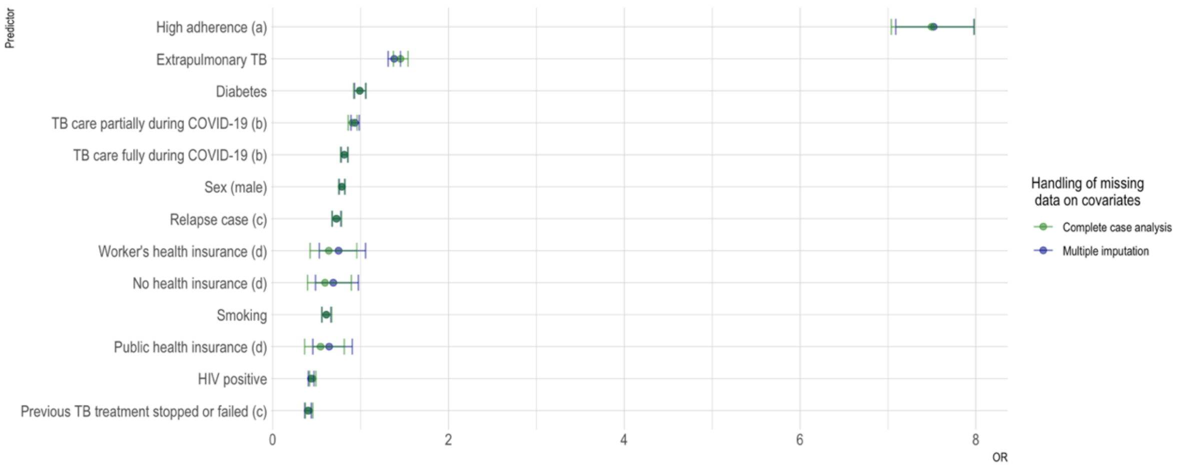

Model 3: Incarcerated

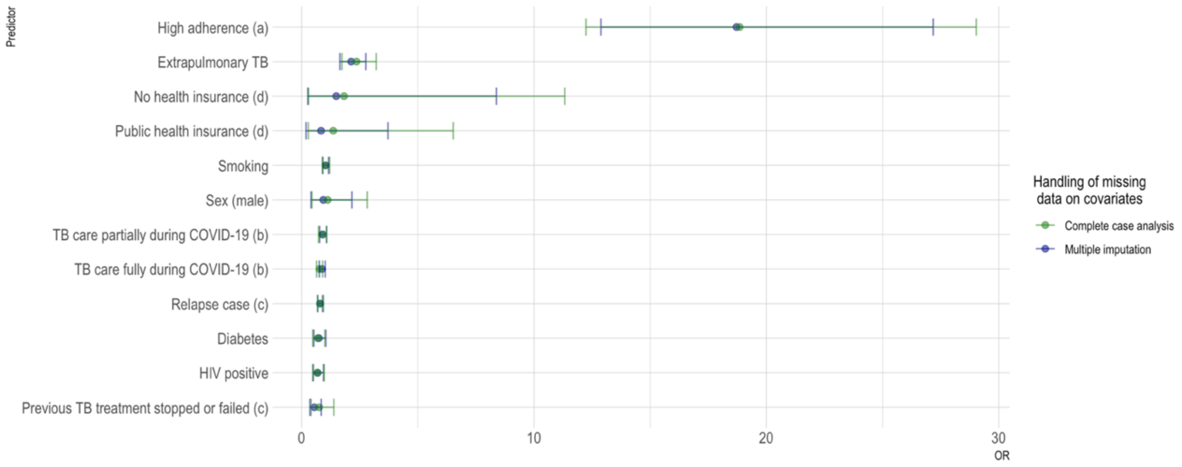

Error bars=95%CI for OR
(a) >=80% of doses taken
(b) Reference category: Pre-COVID-19
(c) Reference category: New TB case
(d) Reference category: Models 1 and 2: Private health insurance. Model 3: Worker's health insurance.

Figure S7. *Comparison of adjusted odds ratios for treatment success under different approaches of handling missing treatment outcome data*

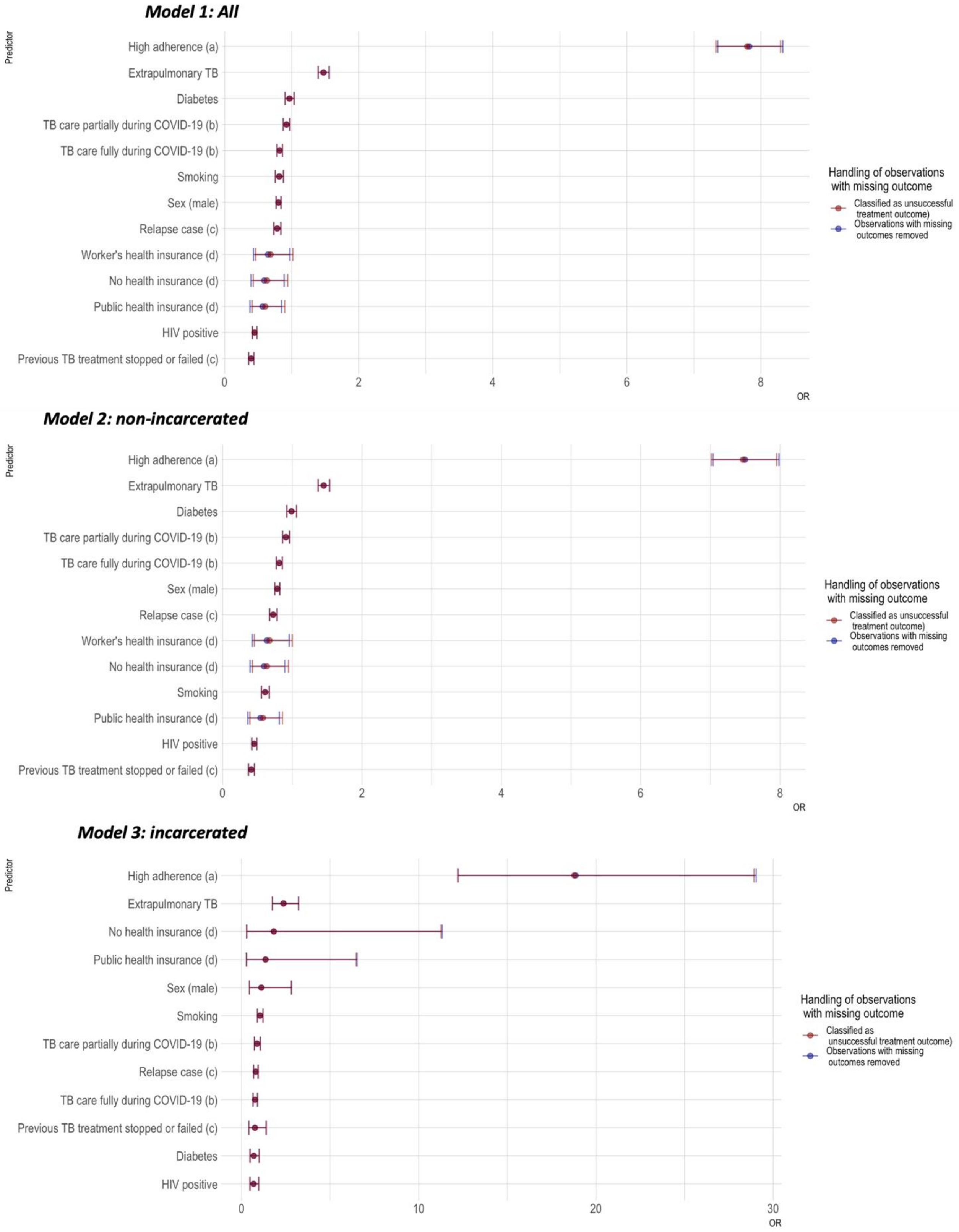

Error bars=95%CI for OR
(a) >=80% of doses taken
(b) Reference category: Pre-COVID-19
(c) Reference category: New TB case
(d) Reference category: Models 1 and 2: Private health insurance. Model 3: Worker's health insurance.

References:

1. Instituto Nacional Penitenciario (INPE). Poblacion penal por situacion juridica y genero segun departamiento. 2023. https://www.inpe.gob.pe/estad%C3%ADstica1.html

2. Instituo Nacional de Estadistica e Informatica. Peru: Estimaciones y proyecciones de población por departamento, provincia y distrito. 2018-2020. 2020. https://www.inei.gob.pe/media/MenuRecursivo/publicaciones_digitales/Est/Lib1715/libro.pdf

3. Instituo Nacional de Estadistica e Informatica. Población estimada y proyectada por sexo y tasa de crecimiento, según años calendarios. 2023. https://m.inei.gob.pe/estadisticas/indice-tematico/population-estimates-and-projections/
