# Supplementary material for: Tuberculosis case notifications and outcomes in Peruvian prisons prior to and during the COVID-19 pandemic: a national-level interrupted time series analysis
Source: Lancet Reg Health Am. 2024 Mar 27;33:100723. doi: 10.1016/j.lana.2024.100723 (PMC11117008; doi:10.1016/j.lana.2024.100723)
Supplement: Spanish_abstract_disclaimer [file mmc2.docx]

*Editorial Disclaimer: This translation in Spanish was submitted by the authors and we reproduce it as supplied. It has not been peer-reviewed. Our editorial processes have only been applied to the original abstract in English, which should serve as a reference for this manuscript.*

**Resumen:**

**Introducción:** La pandemia de COVID-19 ha perturbado significativamente los programas de tuberculosis, por lo que es urgente centrar los esfuerzos de eliminación de la tuberculosis en las poblaciones clave. Las personas encarceladas tienen un alto riesgo de tuberculosis; sin embargo, se desconoce cómo las perturbaciones durante la pandemia de COVID-19 han afectado a las poblaciones encarceladas con tuberculosis.

**Métodos:** Utilizando datos del Programa Nacional de Tuberculosis de Perú de enero de 2018 a diciembre de 2021, se realizó una serie temporal interrumpida de notificaciones de casos de tuberculosis sensible a medicamentos antes y durante la pandemia de COVID-19 (fecha de corte: declaración de emergencia por COVID-19 en Perú, 16 de marzo de 2020). Se exploró mediante regresión logística el efecto de la atención de la tuberculosis que se produjo antes y durante la COVID-19 sobre el éxito del tratamiento de la tuberculosis en las poblaciones encarceladas y no encarceladas.

**Resultados:** Los casos de TB sensible notificados en prisiones desde enero de 2018 hasta diciembre de 2021 (n=10,134) representaron el 10% de todos los casos notificados en el país (n=101,507). En la primera semana de la pandemia de COVID-19, las notificaciones de casos de TB sensible cayeron un 61,2% (IC95%: 59,9-62,7%) en la población no encarcelada y un 17,7% (IC95%: 17,5-17,9%) en la población encarcelada. El éxito del tratamiento de la tuberculosis fue significativamente menor en las personas que recibieron atención de la tuberculosis completamente durante la pandemia de COVID-19 en comparación con los que la recibieron antes de la pandemia de COVID-19 en la población no encarcelada (OR: 0,81; IC del 95 %: 0,78-0,85), pero no fue estadísticamente significativamente menor en la población encarcelada (OR: 0,88; IC95%: 0,76-1,01). El estado de encarcelamiento no modificó el efecto del período de COVID-19 en los resultados del tratamiento de tuberculosis (OR: 1,07, IC del 95%: 0,92-1,25), aunque el éxito del tratamiento fue mayor en la población encarcelada (OR [encarcelados versus no encarcelados, pre -COVID]: 1,52; IC95%: 1,39-1,67).

**Interpretación:** Tanto las poblaciones encarceladas como las no encarceladas experimentaron una gran caída en las notificaciones de casos de TB sensible (aunque mayor en la población no encarcelada). Un menor éxito del tratamiento de la tuberculosis entre los que recibieron atención durante la COVID-19 en la población general indica importantes interrupciones en los servicios de tuberculosis. El hallazgo de que el encarcelamiento en el momento del diagnóstico de tuberculosis se asoció con el éxito del tratamiento es plausible en Perú, dado el aumento de detección de casos y el seguimiento más estricto del tratamiento en las prisiones.

**Financiación:** Canadian Institutes of Health Research (número de referencia de financiación: 179418).
